# Proteolytic Tenderization of Pork Loin with Papain and Bromelain and Its Physicochemical and Sensory Effects

**DOI:** 10.3390/foods15122160

**Published:** 2026-06-15

**Authors:** Mihai Cătălin Ciobotaru, Bianca-Georgiana Anchidin, Diana-Remina Manoliu, Marius Mihai Ciobanu, Paul-Corneliu Boișteanu

**Affiliations:** “Ion Ionescu de la Brad” Iasi University of Life Sciences, 3 Mihail Sadoveanu Alley, 700490 Iasi, Romania; catalin.ciobotaru@iuls.ro (M.C.C.); bianca.anchidin@iuls.ro (B.-G.A.); diana.manoliu@iuls.ro (D.-R.M.); paul.boisteanu@iuls.ro (P.-C.B.)

**Keywords:** meat tenderization, proteolytic enzyme, pork loin, sensory evaluation, meat quality, consumer acceptability, quality attribute, physicochemical property

## Abstract

Improving tenderness in whole-muscle pork products remains a technological challenge, particularly when natural processing strategies are preferred over conventional additives, as texture is regarded as one of the most important quality attributes influencing consumer perception and acceptance of meat products. This study investigated whether two plant proteases, papain and bromelain, incorporated into a red algae-based brine containing Palmaria palmata could enhance the quality of injected pork loin without compromising microbiological safety or sensory acceptance. Seven batches were produced: a control sample and six enzyme-treated samples containing papain or bromelain at 0.015%, 0.030%, and 0.045%. Overall, the enzymatic treatments had a limited effect on proximate composition. However, a modest decrease in fat content was observed, from 3.09% in the control sample to 2.70–2.82% in the samples treated with the highest concentrations of papain and bromelain (0.045%). In contrast, instrumental color and texture were strongly affected. Enzyme-treated samples became lighter, less red, and less saturated, with redness decreasing from 13.07 in the control to 5.19–6.66 in the highest-dose treatments and total color differences reaching 8.66. The most relevant effect was observed in texture, where papain and bromelain markedly reduced shear force, shear work, hardness, gumminess, and chewiness; shear force decreased from 26.22 N/cm^2^ in the control to 10.78 N/cm^2^ and 9.38 N/cm^2^ in the batches treated with the highest enzyme concentrations. During refrigerated storage, total viable counts increased gradually but remained low, with a maximum of 4.56 × 10^2^ CFU/g, while *Escherichia coli*, *Salmonella* spp., and *Listeria monocytogenes* were not detected. Sensory analysis further showed that enzymatic treatment improved perceived tenderness and juiciness without reducing overall acceptability. These findings indicate that papain and bromelain can be used as natural tenderizing tools in injected pork loin, offering a promising route toward cleaner-label meat products with improved texture and preserved microbiological quality.

## 1. Introduction

Meat and meat products are important components of human nutrition, as they provide high biological value proteins, minerals, and essential vitamins [1,2]. Their quality is closely related to nutritional, technological, and sensory attributes, which may be influenced by processing and preservation technologies [3]. In addition, meat quality traits are affected by several intrinsic and extrinsic factors, including muscle characteristics, genotype, diet, muscle location, and environmental conditions [4]. Therefore, strategies aimed at improving the technological and sensory properties of meat products while maintaining nutritional quality and safety remain relevant for meat science and product development [3,5,6].

Before the COVID-19 pandemic, global meat production and consumption were expected to increase by 2030, with global consumption of meat proteins projected to rise by 14%, partly driven by population growth, income growth, and changes in consumer lifestyles [7]. This projected increase in pork consumption has emphasized the importance of developing meat products that meet consumers’ increasingly high-quality requirements [8,9]. In the long term, this trend remains relevant, as global demand for animal-derived products is expected to rise by 2050, in parallel with increasing protein consumption and a world population projected to reach approximately 10 billion people [10]. At the same time, consumers are showing growing concern regarding the nutritional quality of foods, becoming increasingly oriented toward reformulated meat products with a reduced additive content and enriched with bioactive compounds of natural origin [11].

Modern meat processing increasingly uses natural ingredients to support product quality during technological interventions. Red algae-derived compounds may contribute to water retention, texture stability, and nutritional value in meat systems [12,13,14], while seaweeds used as powders or extracts have shown relevant effects on nutritional, textural, and sensory attributes in different food matrices [15,16,17,18]. In this context, their use may be useful as a supportive formulation strategy when enzymatic tenderization is applied since proteases can also generate undesirable textural or sensory changes if not properly controlled. Therefore, physicochemical and sensory evaluation remains essential, particularly because appearance, aroma, texture, and juiciness strongly influence consumer acceptability in meat products [19,20,21,22].

Brine injection technology is widely used in meat processing because it enables the direct distribution of functional ingredients within muscle tissue, improving water-holding capacity, juiciness, and tenderness [23,24]. Compared with traditional marination or tumbling, injection allows for faster and more uniform incorporation of bioactive compounds, enzymes, or natural extracts into the meat structure [25]. In pork loin, this approach has been used to evaluate moisture, color, cooking loss, and texture profile parameters, confirming its relevance for studying physicochemical and textural changes in compact meat cuts [26]. These changes are important because flavor, tenderness, and juiciness are key sensory attributes influencing pork loin quality and overall acceptability [22,27]. Proteolytic enzymes represent an effective strategy for improving meat texture, with exogenous proteases being increasingly studied for obtaining more consistent tenderness. Plant proteases such as papain, actinidin, stem bromelain, zingibain, and ficin/ficain are among the main enzymes used for meat tenderization in different species [28,29].

Plant proteases improve meat tenderness by degrading connective tissue and myofibrillar proteins [30], and enzymes such as papain, bromelain, and ficin are therefore used to optimize meat texture [31]. Their activity over broad pH and temperature ranges supports their technological applicability [29], while strategies such as immobilization, encapsulation, and protein engineering have been explored to improve enzyme stability and activity control [32,33]. However, excessive proteolysis may cause over-tenderization and undesirable sensory changes, as reported for papain and bromelain [34]. For this reason, red algae-derived ingredients may be useful as supportive components due to their water-binding and texture-forming properties. Previous studies showed that κ-carrageenan improved water retention and texture in beef gels, while *Palmaria palmata* powder increased gel hardness and water-holding capacity in meat systems [35,36,37]. Therefore, in this study, the red algae-based brine served as a standardized formulation medium for enzyme incorporation, aimed at mitigating possible undesirable effects of enzymatic tenderization on the meat matrix, while papain and bromelain were evaluated as the main tenderizing variables.

Although papain and bromelain are widely recognized as plant-derived tenderizing enzymes, their comparative use in injected compact whole-muscle pork products remains insufficiently documented. In particular, limited information is available on how enzyme type and dosage influence pork loin quality when physicochemical, textural, microbiological, and sensory parameters are evaluated together. Therefore, the novelty of the present study lies in the direct comparison of papain and bromelain, each applied at three concentrations, 0.015%, 0.030%, and 0.045%, in injected pork loin formulated with a standardized Palmaria palmata-based brine. The algal brine was used as a constant supportive matrix, allowing enzyme type and concentration to be evaluated as the main experimental variables under the same formulation conditions.

Based on this approach, the aim of this study was to assess the influence of papain and bromelain on the quality characteristics of injected pork loin. The samples were analyzed in terms of proximate composition, pH, water activity, instrumental color, Warner–Bratzler shear force, texture profile analysis, microbiological stability during refrigerated storage, and consumer sensory perception. This evaluation was performed to identify the enzymatic treatment that provides the most appropriate balance between tenderness improvement, technological quality, microbiological safety, and sensory acceptability in compact meat products.

## 2. Materials and Methods

### 2.1. Materials

The raw material used in this study was pork loin, represented by the *Longissimus dorsi* muscle, purchased from a slaughterhouse located in Botoșani County, Romania (SC Sagrod SRL). Upon receipt, the raw pork loin was kept under vacuum packaging and stored in refrigerated cabinets at 0–4 °C until processing, in order to prevent quality deterioration and limit microbial development before the manufacture of the experimental batches. Pork loin was selected as the meat matrix because pork is a type of meat in which proteolytic enzymes are frequently applied to improve tenderness and technological quality. Moreover, compared with beef, fewer studies have investigated the effects of proteolytic enzymes on pork meat quality, which supports the relevance of using pork loin in the present study. The pork loin samples were selected from the same commercial batch based on freshness, uniform appearance, absence of visible defects, and similar size in order to minimize variability among samples before processing. Dehydrated red algae (*Palmaria palmata*), originating from the Atlantic Ocean and certified as organic, were purchased from Algamar (Pontevedra, Spain). Papain and bromelain, used as proteolytic enzymes, together with the spices included in the formulation, were purchased from a local company in Iași, Romania (Rocas FDS SRL). Food-grade salt and the other ingredients used for brine preparation were of commercial quality and suitable for meat processing.

### 2.2. Manufacture of Experimental Batches

Upon receipt, the raw pork loin was stored under refrigerated conditions at 0–4 °C to prevent quality deterioration before processing. The meat pieces were selected based on freshness, color uniformity, fat level, and structural integrity. Samples showing visible defects or undesirable size variations were excluded. Subsequently, the selected pork loin pieces were trimmed by removing visible connective tissue and excess surface fat in order to standardize the raw material and optimize the subsequent processing steps.

#### 2.2.1. Brine Preparation

For the production of the experimental batches, a fresh brine containing 18% NaCl was prepared. The control batch (LCT-0) was formulated with a brine based on water, sodium chloride, and red algae (*Palmaria palmata*) extract, but without the addition of proteolytic enzymes. This formulation allowed the specific effects of papain and bromelain to be evaluated against a non-enzymatic control containing the same red algae-based brine.

The brine ingredients were dissolved in water using a double-jacketed mixer, which maintained the solution temperature between 0 and 4 °C in order to limit microbial proliferation during preparation. First, the required volume of water was measured, and sodium chloride was gradually dissolved until a homogeneous solution was obtained. Subsequently, the dehydrated *Palmaria palmata* biomass was rehydrated in 500 mL of the previously prepared brine for 10 min, according to the manufacturer’s recommendations. After rehydration, the algae biomass was mechanically homogenized until a fine and uniform suspension was obtained. The suspension was then filtered to remove residual particles, and the resulting red algae extract was gradually incorporated into the main brine under continuous stirring to ensure uniform dispersion throughout the solution.

The final stage of brine preparation involved the addition of proteolytic enzymes according to the concentrations established for each experimental batch.

The concentration of ingredients incorporated into the brine was determined by mass balance, taking into account both the injection level and the target concentration in the injected product. Brine injection was performed at 10% of the raw pork loin weight, corresponding to an injection level of p = 0.10. Accordingly, the mass of brine added was calculated using Equation (1):

(1) B=p×M

where B represents the mass of brine added, p represents the injection level, and M represents the initial raw meat mass.

The final mass of the injected product was calculated using Equation (2):

(2) $$M_{f}=M(1+p)$$

where $M_{f}$ represents the final mass of the injected product.

Under these conditions, the final concentration of each additive in the injected product was calculated using Equation (3):

(3) $$C_{f\;}=\;\frac{C_{b}\times p}{1+p}$$

where $C_{f\;}$ represents the final concentration of the additive in the injected product, and $C_{b}$ represents the concentration of the additive in the brine.

By rearranging Equation (3), the concentration required in the brine was calculated using Equation (4):

(4) $$C_{b}=C_{f}\times \frac{1+p}{p}$$

Based on Equation (4), the brine was formulated to obtain 1.00% red algae (*Palmaria palmata*) and 0.015%, 0.030%, and 0.045% papain or bromelain in the injected product, and the corresponding calculated concentrations in the brine are presented in Table 1.

The complete brine mixture was homogenized in the mixer to ensure the uniform dispersion of the enzymes and red algae extract throughout the solution. This step was performed under strict temperature control in order to prevent the degradation or denaturation of bioactive compounds and to maintain the stability of the final brine.

#### 2.2.2. Brine Injection, Tumbling, and Thermal Processing

Brine injection was performed automatically using an Inwestpol A-10 injector (Gdańsk, Poland). The equipment was fitted with 10 stainless-steel needles, uniformly arranged to ensure homogeneous brine distribution throughout the pork loin pieces. The injector pump was adjusted to maintain a constant pressure of 1.5 bar, in order to prevent excessive damage to the muscle fibers and minimize brine loss during injection.

After injection, the samples were subjected to tumbling to facilitate and homogenize brine diffusion within the pork loin matrix. This step was carried out using an Inwestpol MAL 200/300 tumbler (Gdańsk, Poland) equipped with vacuum and a rotating drum. After tumbling, the pork loin pieces were manually tied with food-grade string and uniformly placed on stainless-steel rods mounted on the processing trolley, ensuring that the samples did not touch each other and allowing for uniform air circulation during thermal processing. The samples were then introduced into the thermal processing chamber (Stawiany, Pszczółki, Poland). The thermal processing stages and parameters applied to all seven pork loin batches are presented in Table 2.

The initial drying stage aimed to remove surface moisture from the pork loin samples, facilitating the formation of a surface layer that favored uniform smoke deposition. The smoking process was carried out using cherry wood, selected for its moderate smoke generation and aromatic profile, which is suitable for pork products and contributes to the sensory characteristics of the final product.

After smoking, the samples were subjected to thermal treatment in a technological chamber until a minimum core temperature of 72 °C was reached in order to ensure product safety and comply with the hygiene requirements established by Regulation (EC) No. 852/2004 [38].

The final drying stage was performed as the last step of thermal processing to reduce excess surface moisture and improve the structural stability of the product. By promoting the formation of a firm and uniform surface, this stage contributed to maintaining the texture and sensory quality of the pork loin samples during refrigerated storage.

### 2.3. Analytical Methods

#### 2.3.1. Physicochemical Analysis

The physicochemical content of the pork loin samples was evaluated using conventional procedures for meat and meat products. Moisture content was evaluated by oven drying, as indicated in ISO 1442:2023 [39]. Briefly, homogenized materials were weighed before and after drying, and moisture content was determined using the mass loss measured after thermal dehydration. The dry matter content was estimated by subtracting moisture (%) from 100, according to the following equation: Dry matter (%) = 100 − moisture (%).

The ash content was measured by incineration in a muffle furnace in accordance with ISO 936:1998 [40]. The samples were mineralized at high temperatures until the organic matter was completely combusted, and the ash content was reported as a percentage of the original sample mass. Protein content was assessed using the Kjeldahl reference technique, as given in ISO 937:2023 [41], and total protein was estimated using the appropriate nitrogen-to-protein conversion factor for meat products. The fat content of meat and meat products was determined using the reference technique defined in ISO 1443:1973 [42].

Carbohydrate content was calculated by difference using the following equation: Carbohydrates (%) = 100 − [moisture (%) + protein (%) + fat (%) + ash (%)]. The energy value was calculated using the general Atwater conversion factors, considering 4 kcal/g for protein, 9 kcal/g for fat, and 4 kcal/g for carbohydrates [43].

Water activity (a_w_) was measured instrumentally using an AquaLab 4TE analyzer (Addium Inc., Washington, DC, USA). Sample pH was determined by direct insertion of a meat-specific penetration electrode connected to a digital pH meter (HI98163, Hanna Instruments, Nușfalău, Romania). The instrument was calibrated prior to analysis with standard buffer solutions at pH 4.01 and 7.01. Measurements were performed after temperature equilibration, and the final pH value was recorded only after signal stabilization.

#### 2.3.2. Instrumental Colorimetric Analysis

The color of the pork loin samples was measured using a portable Konica Minolta CR-410 colorimeter (Konica Minolta Inc., Tokyo, Japan) within the CIE L*a*b* color space. The measurements were performed using illuminant D65, a 10° standard observer angle, and a 50 mm measuring aperture. Before analysis, the instrument was calibrated using a white calibration plate, according to the manufacturer’s instructions. Five readings were taken at different surface points of each sample to account for color variability and were used as individual observations in the statistical analysis.

Chroma (C*) and hue angle (h*) were calculated using Equations (5) and (6) [44,45]. In Equation (6), *tan*^−1^ denotes the inverse tangent function, also known as arctangent, while a* and b* represent the chromatic coordinates of the CIELAB color space.

(5) $$\mathrm{Chroma}\;(C^{*})=\sqrt{{\left(a^{*}\right)}^{2}+{\left(b^{*}\right)}^{2}}$$

(6) $$\mathrm{Hue}\;\mathrm{angle}\;(h^{*})={tan}^{-1}(b^{*}/a^{*})$$

Total color difference (Δ*E*) was used to quantify the overall color variation between the enzyme-treated samples and the control sample, considering the combined changes in L*, a*, and b* coordinates [46]. The Δ*E* values were calculated using Equation (7) [44]:

(7) $$\Delta E=\sqrt{\;{{(a}^{*})}^{2}+({{\Delta b}^{*})}^{2}+({{\Delta L}^{*})}^{2}\;}$$

#### 2.3.3. Instrumental Texture Analysis

Instrumental texture analysis was performed to evaluate the mechanical properties and tenderness of the pork loin samples. The measurements were carried out using a TA1+1K Plus texture analyzer (Ametek Inc., Berwyn, PA, USA) equipped with a 500 N load cell. Two types of tests were applied: Warner–Bratzler shear force analysis and Texture Profile Analysis (TPA).

For the shear test, cylindrical samples were prepared with a length of 7 cm and a diameter of 15 mm. The samples were cut using a Warner–Bratzler V-shaped blade, and the parameters recorded were shear force (N) and shear work (mJ). These parameters were used to evaluate the resistance of the samples to cutting and the mechanical energy required for sample sectioning.

Texture Profile Analysis was performed using a cylindrical POM/Delrin probe with a diameter of 25 mm. The samples were subjected to a double-compression test, and the following parameters were recorded: hardness (N), adhesiveness (N·s), cohesiveness, springiness, gumminess (N), and chewiness (N·mm).

To ensure reproducibility and comparability among samples, all texture determinations were carried out under identical testing conditions, with the same speed and deformation settings applied throughout the analysis.

#### 2.3.4. Microbiological Analyses

The microbiological quality of the pork loin samples was evaluated to assess both the hygienic status of the product and its microbiological safety during refrigerated storage. Total viable count was determined as a general indicator of microbial load and hygienic quality, while *Escherichia coli* was analyzed as an indicator of possible fecal contamination or inadequate hygiene during processing. In addition, *Listeria monocytogenes* and *Salmonella* spp. were investigated because these pathogens are not permitted in ready-to-eat meat products and their detection is required to verify product safety and compliance with microbiological food safety criteria.

Samples were analyzed at three storage times: day 0, day 7, and day 14. The selection of days 7 and 14 as microbiological sampling points is consistent with previous meat product studies, such as that of [47], in which refrigerated storage intervals of 0, 7, and 14 days were used to monitor microbial development and assess product stability over time.

The microbiological determinations were performed according to the ISO reference methods specific to each tested microbial group or pathogen. The results were interpreted in accordance with the criteria established by Regulation (EC) No. 2073/2005 on microbiological criteria for foodstuffs [48].

- Sample Preparation and Serial Dilutions

For each sample, 1 g of product was aseptically collected and homogenized with 9 mL of sterile peptone water, obtaining the initial 10^−1^ dilution. The samples were then vortexed to ensure homogenization, and additional serial dilutions were prepared up to 10^−3^. However, only the first dilution (10^−1^) was used for result reporting, as the microbial counts obtained from this dilution were within the appropriate range for interpretation.

- Total Viable Count (TVC)

Total viable count (TVC) was determined by plating the appropriate dilution on Plate Count Agar (PCA) medium (Scharlau, Barcelona, Spain), according to ISO 4833-1:2013 [49]. The plates were incubated at 30 °C for 72 ± 3 h, after which the developed colonies were counted manually. The results were calculated as colony-forming units per gram of sample and expressed as ×10^2^ CFU/g. TVC was used as an indicator of the overall hygienic quality of the samples and of the evolution of viable microbiota during refrigerated storage.

- Enumeration of *Escherichia coli*

Detection and enumeration of Escherichia coli were performed according to ISO 16649-2:2001 [50], using RAPID’ *E. coli* 2 Agar medium (Bio-Rad, Marnes-la-Coquette, France). The inoculated plates were incubated at 44 ± 1 °C for 24 ± 2 h. After incubation, typical colonies were counted, and the results were expressed as colony-forming units per gram of sample (CFU/g).

- Detection of *Listeria monocytogenes*

Detection of *Listeria monocytogenes* was performed according to ISO 11290-1:2017 [48]. The analysis was carried out using RAPID’ *Listeria* spp. medium (Bio-Rad, Marnes-la-Coquette, France), and the presence of *L. monocytogenes* was assessed in 25 g of sample. The results were expressed as detected/not detected in 25 g.

- Detection of *Salmonella* spp.

Detection of *Salmonella* spp. was performed according to ISO 6579-1:2017 [51]. The samples were enriched using selective enrichment media, including Rappaport–Vassiliadis soya broth (RVS) and Muller–Kauffmann tetrathionate–novobiocin broth (MKTTn), followed by plating on RAPID’*Salmonella* Agar medium (Bio-Rad, Marnes-la-Coquette, France). The presence of *Salmonella* spp. was assessed in 10 g of sample, and the results were expressed as detected/not detected in 10 g. Compliance was evaluated based on the absence of *Salmonella* spp. in the analyzed sample amount.

#### 2.3.5. Consumer Sensory Analysis

Sensory evaluation was performed in two sessions with the same panelists, following the general principles described in ISO 8586:2023 [52], ISO 8589:2007 [53], and ISO 13299:2016 [54]. The analysis was carried out in a dedicated sensory laboratory at the “Ion Ionescu de la Brad” Iași University of Life Sciences, equipped with individual testing booths, neutral lighting (6500 K), and controlled ambient temperature (21 ± 1 °C).

The sensory panel consisted of 30 semi-trained assessors aged between 25 and 43 years, selected based on their willingness to consume meat products, ability to distinguish basic sensory attributes, and previous participation in similar sensory evaluation sessions [55]. Before the evaluation, all participants signed a voluntary participation agreement and received instructions regarding the evaluation procedure. The instructions also included explanations regarding the use of the sensory scales, the meaning of the sensory descriptors, and the interpretation of the scale anchors.

The first session was used to familiarize the assessors with the pork loin samples and to verify the relevance of the sensory descriptors included in the lexicon developed for this product category [56,57]. During this session, the assessors were also instructed on the consistent interpretation of the selected descriptors and on the correct use of the scoring criteria. After this preliminary session, the final descriptors listed in Table 3 were selected and used for sample evaluation.

In the second session, all seven pork loin batches, corresponding to all experimental treatments, were evaluated by the same panelists. The samples were not divided across multiple evaluation sessions; instead, all treatments were assessed during the same evaluation session. The samples were sliced into uniform portions, coded with random three-digit numbers, and served in randomized order. Water was provided for palate cleansing between samples.

The sensory evaluation included a hedonic test for overall acceptability, a CATA test based on the descriptors listed in Table 3, and a Quantitative Descriptive Analysis (QDA) for assessing the intensity of selected sensory attributes. Overall acceptability was evaluated using a 9-point hedonic scale, ranging from strong dislike to strong liking [58,59]. Specifically, the hedonic scale was anchored as follows: 1 = “dislike extremely”, 2 = “dislike very much”, 3 = “dislike moderately”, 4 = “dislike slightly”, 5 = “neither like nor dislike”, 6 = “like slightly”, 7 = “like moderately”, 8 = “like very much”, and 9 = “like extremely”. The intensity of sensory attributes was assessed using a 9-point structured scale, where 1 indicated an absent or very weak perception and 9 indicated a very strong perception. For this structured intensity scale, a score of 1 indicated absent or very weak perception, scores of 2–3 indicated weak intensity, scores of 4–6 indicated moderate intensity, scores of 7–8 indicated high intensity, and a score of 9 indicated very strong intensity.

After evaluation, CATA data were used to identify differences among samples based on descriptor selection, QDA data were used for Principal Component Analysis (PCA), and PREFMAP analysis was applied to relate consumer preference data to the sensory profile of the samples [60,61].

### 2.4. Statistical Analysis

All analyses were performed in five replicates, and the results were expressed as mean ± standard deviation (SD). Statistical analysis of the physicochemical, colorimetric, textural, and microbiological data was performed using IBM SPSS Statistics software, version 26.0 (IBM Corp., Armonk, NY, USA). One-way analysis of variance (ANOVA), followed by Tukey’s post hoc test, was applied to identify significant differences among the experimental batches. Differences were considered statistically significant at *p* < 0.05.

In addition, the effects of enzyme type and enzyme concentration on the analyzed parameters were evaluated separately using two-way analysis of variance, and the corresponding *p*-values were reported in the tables to highlight the individual influence of these two experimental factors. Sensory data analysis, including CATA data processing, Principal Component Analysis (PCA), PREFMAP analysis, and graphical visualization, was performed using XLSTAT software, version 2025.1 (Addinsoft, Paris, France).

## 3. Results and Discussion

### 3.1. Physicochemical Analysis of the Meat Products

The enzymatic treatments induced only moderate changes in the chemical composition of the pork loin samples, while the main proximate fractions remained relatively stable (Table 4). Dry matter and moisture varied within narrow ranges, from 25.56 to 25.84% and from 74.16 to 74.44%, respectively. Enzyme type significantly influenced these parameters (*p* = 0.039), whereas enzyme concentration had no significant effect (*p* = 0.102). The limited variation observed for moisture and dry matter indicates that the treatments did not markedly alter the basic compositional profile of the product. This result can be discussed in relation to the constant presence of *Palmaria palmata* in the injection brine, as seaweed-derived fibers and polysaccharides have been associated with water-binding and water-holding properties in meat products [62,63,64].

Water activity remained practically unchanged among the samples, with values ranging from 0.97 to 0.98, and was not significantly affected by enzyme type or concentration (*p* > 0.05). Therefore, papain and bromelain did not reduce free-water availability in the meat matrix. The microbiological stability of the samples should consequently be attributed mainly to thermal treatment, hygienic processing, and refrigerated storage, rather than to changes in a_w_ [65].

Fat content showed the clearest compositional response to enzyme concentration. The highest value was recorded in the control sample, LCT-0, while lower values were observed in the enzyme-treated samples, especially LPAP-3 and LBRO-3. Enzyme concentration had a significant effect on fat content (*p* = 0.003), whereas enzyme type was not significant (*p* = 0.064). In agreement with this trend, energy value decreased from 113.21 kcal/100 g in the control sample to 109.49 kcal/100 g in LPAP-3 and 110.54 kcal/100 g in LBRO-3. This reduction was significantly influenced by both enzyme type (*p* = 0.043) and enzyme concentration (*p* = 0.002), and can be mainly explained by the lower lipid content of the treated samples, since fat is the major contributor to caloric density in meat products.

Protein content remained relatively constant, ranging from 21.01 to 21.32%, and was not significantly affected by enzyme type or concentration (*p* > 0.05). These results indicate that papain and bromelain did not substantially modify the total protein content measured by proximate analysis. Instead, the observed quality changes should be discussed in relation to structural modifications in meat proteins, as plant proteases have been reported to hydrolyze myofibrillar proteins into lower-molecular-weight fractions [30]. Similar observations were reported by Aziz et al. [66] and Gil et al. [67], who found that proteolytic treatments may affect meat quality attributes without necessarily inducing major changes in total protein content.

Ash and carbohydrate contents showed limited variations. Although both parameters were numerically higher in the enzyme-treated samples than in the control, neither enzyme type nor enzyme concentration had a significant effect (*p* > 0.05). These differences therefore appear to reflect the contribution of the injected formulation rather than a specific effect of papain or bromelain.

The pH values were significantly affected by both enzyme type (*p* < 0.0001) and enzyme concentration (*p* = 0.012). The highest values were recorded in the bromelain-treated samples, particularly LBRO-2 and LBRO-3, while the papain-treated samples remained closer to the control. The higher pH values observed in the bromelain-treated samples indicate an enzyme-dependent effect under the conditions of the present study. Although plant proteases are known to act on myofibrillar and connective tissue proteins during meat tenderization [30], the present results do not allow for a direct attribution of pH changes to proteolysis.

### 3.2. Instrumental Color Analysis

The colorimetric profile of the pork loin samples was significantly affected by enzymatic treatment with papain and bromelain (Table 5). Enzyme type significantly influenced L* (*p* = 0.001), a* (*p* < 0.0001), C* (*p* < 0.0001), h* (*p* < 0.0001), and Δ*E* (*p* < 0.0001), whereas its effect on b* was not significant (*p* = 0.219). Enzyme concentration significantly affected all color parameters, indicating that the chromatic response depended not only on the protease used, but also on treatment intensity.

Lightness increased in all enzyme-treated samples compared with the control, with L* values ranging from 68.67 ± 0.62 in LCT-0 to 74.25 ± 0.31 in LPAP-2 and 74.25 ± 0.96 in LBRO-3. The increase in L* values indicates that enzymatic treatment modified the optical characteristics of the pork loin samples. Similar relationships between higher L* values and moisture-related changes in meat have been reported by Qiao et al. [68].

The most pronounced color modification was observed for redness. The a* value decreased from 13.07 ± 0.39 in the control sample to 5.19–8.45 in the treated samples, with the lowest value recorded in LPAP-3. This reduction, together with the decrease in C* from 15.47 ± 0.28 in the control to 10.19–12.64 in the treated samples, indicates a loss of red color intensity and saturation after enzymatic treatment. These changes are consistent with the structural effects of proteolytic enzymes on muscle tissue, including collagen solubilization, myofibrillar fragmentation, and alteration in the muscle matrix, as previously reported for papain-treated meat systems [69].

The b* parameter showed a moderate increase in most treated samples, reaching the highest value in LBRO-3 (9.63 ± 0.06), and was significantly influenced by enzyme concentration (*p* < 0.0001), but not by enzyme type. A comparable non-linear behavior of b* during papain and bromelain treatment was reported by Aziz et al. [66], although without significant differences among treatments. In the present study, the simultaneous decrease in a* and increase in b* contributed to the rise in h* values, from 0.56 ± 0.02 in the control to 0.81–1.04 in the treated samples. According to Ruedt et al. [70], hue angle describes the position of color within the chromatic space. In the present study, the higher h* values indicated a shift from a redder appearance toward a more yellowish tone, especially at the highest enzyme concentrations.

The overall color difference, expressed as Δ*E*, ranged from 5.13 ± 0.60 to 8.66 ± 0.35 compared with the control. The lowest ΔE values were recorded for LBRO-1 and LBRO-2. These results indicate smaller overall color differences from the control at low and medium bromelain concentrations. In contrast, the highest Δ*E* values were observed for LBRO-3, LPAP-1, and LPAP-3, indicating more evident changes in the overall color profile.

Overall, papain-treated samples showed lower a* and C* values than bromelain-treated samples. This indicates a stronger reduction in redness and color saturation in the papain-treated groups. These color differences are consistent with previous reports showing that plant cysteine proteases can affect meat protein structure and quality attributes. However, the present study did not directly assess structural protein degradation; therefore, the color changes should be interpreted as treatment-dependent effects. Ha et al. [71] reported that commercial papain and bromelain formulations are among the most active proteases tested on meat proteins, while Gagaoua et al. [29] emphasized that their use requires careful optimization to avoid undesirable effects on quality attributes other than tenderness. Differences among studies may be explained by meat species, muscle structure, enzyme concentration, exposure time, processing conditions, and thermal treatment [29,69].

### 3.3. Texture Profile Analysis and Shear Properties

The results presented in Table 6 show that enzymatic treatment with papain and bromelain markedly modified the textural profile of the pork loin samples. Enzyme concentration had a significant effect on Warner–Bratzler shear force, work of shear, hardness, springiness, gumminess, chewiness, and adhesiveness (*p* < 0.05), whereas enzyme type was not significant for most parameters, except adhesiveness (*p* < 0.0001). These results indicate that enzyme concentration was the main experimental factor associated with the observed textural changes. This interpretation is consistent with previous studies showing that tenderization by plant proteases depends on enzyme dose, exposure time, enzymatic activity, and muscle matrix characteristics [30,72].

Warner–Bratzler shear force decreased from 26.22 N/cm^2^ in the control sample to 10.65–16.15 N/cm^2^ in the papain-treated samples and 9.38–13.55 N/cm^2^ in the bromelain-treated samples. Similarly, work of shear decreased from 602.50 mJ in LCT-0 to 210.71–352.95 mJ in the papain-treated groups and 245.02–303.38 mJ in the bromelain-treated groups. These reductions indicate that the enzyme-treated samples required less force and less mechanical energy for sectioning, reflecting lower structural resistance. The decrease in shear force and shear work is consistent with the tenderizing effect of papain and bromelain reported in previous studies [30,31,73,74,75].

The same trend was observed for TPA parameters associated with firmness and mastication. Hardness decreased from 41.29 N in the control to 15.35–28.67 N in the papain-treated samples and 18.49–20.76 N in the bromelain-treated samples. Gumminess and chewiness also decreased markedly, with the lowest values generally recorded in the samples treated with the highest enzyme concentrations, especially LPAP-3 and LBRO-3. These changes indicate a weakening of the muscle protein network and improved chewability of the samples. Similar reductions in hardness and mastication-related parameters after proteolytic treatment have been reported in meat and fish matrices treated with papain or bromelain [30,72,74,76,77,78,79].

Cohesiveness was the only textural parameter not significantly affected by enzyme concentration (*p* > 0.05), despite numerical decreases in most treated samples. This suggests that enzymatic treatment reduced firmness and mechanical resistance more clearly than it affected the ability of the matrix to maintain internal structural integrity. Such behavior has also been reported in enzyme-treated meat products, where hardness may decrease without a proportional reduction in cohesiveness [72,78].

Springiness decreased from 0.26 in the control sample to 0.11–0.16 in the papain-treated groups and 0.09–0.14 in the bromelain-treated groups, indicating a reduced ability of the treated samples to recover their original shape after compression. Adhesiveness showed a different pattern, being the only parameter significantly influenced by enzyme type (*p* < 0.0001), with lower values in the bromelain-treated samples than in the papain-treated samples. This result indicates that adhesiveness was more dependent on enzyme type than the other texture parameters. Previous studies have shown that papain and bromelain can generate different textural responses depending on substrate, dose, and processing conditions [30,71,72].

Overall, enzymatic treatment improved the tenderness-related characteristics of pork loin, as shown by the concomitant decreases in Warner–Bratzler shear force, work of shear, hardness, gumminess, and chewiness. The strongest textural changes were generally observed in LPAP-3 and LBRO-3, confirming the key role of enzyme concentration in modulating the final texture. Since total protein content remained relatively constant among samples (Table 4), the effects of papain and bromelain should be interpreted mainly as structural modifications in muscle proteins rather than as a reduction in total protein content. However, direct biochemical confirmation of protein degradation, such as SDS-PAGE, peptide profiling, soluble protein determination, or free amino acid analysis, was not performed and should be considered in future studies.

### 3.4. Microbiological Analysis of Meat Products

Table 7 presents the microbiological evolution of pork loin samples during refrigerated storage. The total viable count (TVC) increased gradually in all experimental batches from D0 to D14, indicating the expected microbial development in meat products stored under refrigerated conditions [80]. In the control sample, TVC increased from 0.51 ± 0.04 × 10^2^ CFU/g on D0 to 1.62 ± 0.14 × 10^2^ CFU/g on D14, whereas the enzyme-treated samples showed higher values, particularly at the highest enzyme concentrations. On D14, the highest TVC values were recorded in LBRO-3 and LPAP-3, reaching 4.56 ± 0.03 × 10^2^ CFU/g and 4.07 ± 0.02 × 10^2^ CFU/g, respectively. Similar increases in microbial load during storage were reported by Akkaya et al. [79] in beef samples treated with papain and bromelain during cold aging.

The enzyme-treated samples showed higher TVC values than the control, particularly at the highest enzyme concentrations. Previous studies reported that plant proteases can generate low-molecular-weight compounds and free amino acids [81], and that such substances may support the growth of spoilage microorganisms more readily than intact meat proteins [82]. In the present study, however, the biochemical compounds generated during enzymatic treatment were not directly quantified. Therefore, the TVC increase should be interpreted as a treatment-dependent microbiological evolution during refrigerated storage, rather than as direct evidence of proteolysis-derived substrate formation.

Water activity and pH also supported microbial development during storage. In the present study, water activity remained high, between 0.97 and 0.98, indicating substantial water availability within the meat matrix. Foods with water activity above 0.95 provide sufficient moisture for the growth of bacteria, yeasts, and molds [83], and meat with high water activity and pH values around or below 6.0 may provide favorable conditions for microbial development [84]. Thus, papain and bromelain cannot be considered microbiological stabilization strategies per se, as they did not reduce water activity to inhibitory levels. Under these conditions, the progressive increase in TVC from D0 to D14 can be discussed in relation to residual microbiota, available water, and permissive pH. The possible contribution of readily metabolizable substrates should be interpreted cautiously, as these compounds were not directly quantified in the present study.

Nevertheless, the absolute TVC levels remained low throughout storage. The maximum value recorded, 4.56 × 10^2^ CFU/g in LBRO-3 on D14, corresponding to approximately 2.66 log10 CFU/g, was substantially below the levels commonly associated with microbiological spoilage of pork [85]. Therefore, although enzymatic treatment was associated with increased TVC values, the results do not indicate advanced microbiological deterioration during the examined storage period.

*Listeria monocytogenes*, *Salmonella* spp., and *Escherichia coli* were not detected in any sample at D0, D7, or D14. This confirms the appropriate hygienic-sanitary status of the samples and indicates that the applied treatments did not compromise microbiological safety. From a legislative perspective, Regulation (EC) No. 2073/2005 establishes microbiological criteria for foodstuffs, including requirements for *Listeria monocytogenes* and *Salmonella* spp., depending on the product category and its ability to support pathogen growth [48]. Under the investigated experimental conditions, the absence of these microorganisms confirmed the microbiological compliance of the pork loin samples during refrigerated storage.

Overall, papain and bromelain influenced TVC development in refrigerated pork loin samples, with the effect depending on enzyme type and concentration. However, the observed increase should be interpreted as a controlled microbial evolution under refrigerated storage rather than as evidence of microbiological deterioration, since pathogen indicators remained negative and TVC values stayed at low levels.

### 3.5. Sensory Evaluation

#### 3.5.1. Hedonic Evaluation of Overall Acceptability

**Figure 1:**
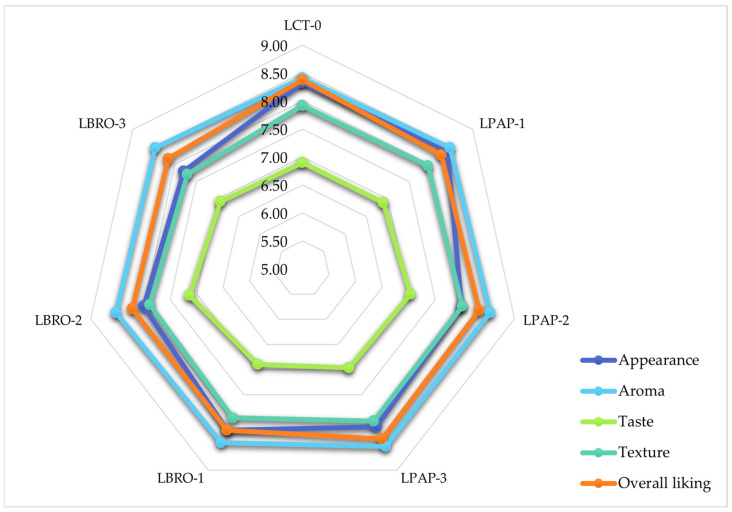
Radar plot of the mean hedonic scores for appearance, aroma, taste, texture, and overall liking of the control sample and samples treated with papain and bromelain at different concentrations. LCT-0—control sample; LPAP-1, LPAP-2, and LPAP-3—samples treated with papain at 0.015%, 0.030%, and 0.045%, respectively; LBRO-1, LBRO-2, and LBRO-3—samples treated with bromelain at 0.015%, 0.030%, and 0.045%, respectively.

Regarding the hedonic evaluation of the analyzed samples (Figure 1), similarities were observed between the enzyme-treated samples (LPAP-1, LPAP-2, LPAP-3, LBRO-1, LBRO-2, and LBRO-3) and the control sample (LCT-0) for attributes such as aroma, taste, and overall liking. The sensory attributes appearance and texture showed greater variation in mean scores among the analyzed samples.

Overall, the enzyme-treated samples obtained slightly higher mean scores than the control sample, indicating that the inclusion of papain and bromelain improved consumers’ hedonic perception.

Although the aroma of the samples showed similar mean values among the studied groups, slightly higher scores were recorded in the enzyme-treated samples, especially at the 0.030% enzyme concentration (LPAP-2 and LBRO-2), for both enzyme types analyzed. However, beyond this level, a slight decrease in the mean aroma scores was observed as the enzyme concentration increased to 0.045%.

The appearance of the samples varied to a greater extent among the enzyme-treated pork loin samples, with higher scores being obtained in the groups treated with 0.015% enzyme (LPAP-1 and LBRO-1), even compared with the control sample (LCT-0). However, as enzyme concentration increased, sample appearance was slightly affected, as the mean scores decreased in the groups treated with 0.030% (LPAP-2 and LBRO-2) and 0.045% enzyme (LPAP-3 and LBRO-3), reaching values even below the overall mean score of the control sample (LCT-0). Nevertheless, overall liking was not negatively affected, as the mean scores for this parameter remained relatively constant.

Similarly to aroma, the taste of the samples showed a positive increasing trend following enzyme addition, for both papain and bromelain, reaching the highest scores at the 0.030% enzyme concentration (LPAP-2 and LBRO-2), which exceeded all other analyzed samples. However, a further increase in enzyme concentration to 0.045% (LPAP-3 and LBRO-3) led to a slight decrease in taste quality, this effect being more noticeable in the sample treated with 0.045% bromelain (LBRO-3).

As observed for taste, the hedonic perception of texture also recorded higher mean scores when enzyme concentration increased up to 0.030% (LPAP-2 and LBRO-2), compared with the control sample (LCT-0). However, as in the case of taste, increasing the enzyme concentration to 0.045% led to a slight decrease in the positive perception of texture in the sample treated with 0.045% bromelain (LBRO-3), compared with the papain-treated sample (LPAP-3), whose hedonic texture score remained relatively constant.

Overall, the hedonic profile shows that the sensory perception of the analyzed samples improved slightly but consistently as a result of the enzymes used, with the best results being recorded at the intermediate addition level. Increasing the enzyme concentration to 0.045% was associated with a slight decrease in hedonic scores for certain attributes, suggesting that the 0.030% concentration represented the level at which the sensory benefits were most clearly expressed.

The results reported by Bhattarai and Lamichhane [86] are consistent with the trend observed in the present study, characterized by slightly higher hedonic scores for the enzymatically treated samples and by the identification of an intermediate enzyme addition level as the most favorable. Using concentrations of 0, 10, 20, 40, 60, 80, 90, and 100 mg/L for papain and bromelain, the authors treated buffalo meat intended for sukuti production by injecting an enzyme solution at a concentration of 10% w/w. Texture, color, aroma, and overall acceptability were evaluated using a 9-point hedonic scale.

Overall, enzyme-treated samples showed higher hedonic acceptance than the control sample, both in the present study and in the work reported by Bhattarai and Lamichhane [86], indicating that the use of plant enzymes may improve the overall sensory impression of the product. However, the hedonic response differed between the two enzymes. In the aforementioned study, the most favorable sensory evaluation for bromelain was obtained at the lower level of 10 mg/L (~0.0001%), whereas the optimal level for papain was an intermediate concentration of 40 mg/L (~0.0004%). Both treatments outperformed the comparable control samples. Thus, while in the case of papain the best sensory evaluation was associated with an intermediate treatment level, for bromelain a lower level was sufficient to exceed the control sample. Higher concentrations became less advantageous, most likely due to excessive softening of the texture, as noted by the authors. This tendency was also evident in the present study, where the sample treated with 0.045% bromelain (LBRO-3) recorded the lowest mean hedonic texture score among all analyzed variants. Similarly, increasing the papain concentration to 0.045% (LPAP-3) led to a slight reduction in the sensory perception of texture, although this decrease was less pronounced than that observed for the sample treated with the same concentration of bromelain.

These results suggest that increasing enzyme addition beyond a certain threshold no longer provides additional sensory benefits and may even induce negative effects, especially when an excessively tender texture develops.

#### 3.5.2. CATA Analysis

Table 8 presents the *p*-values obtained using Cochran’s Q test, together with the selection frequencies of the CATA attributes for each of the examined samples (LCT-0, LPAP-1, LPAP-2, LPAP-3, LBRO-1, LBRO-2, and LBRO-3). Cochran’s Q test indicated that, among the evaluated attributes, only “Presence of a white layer” showed statistically significant differences among samples (*p* = 0.0009), whereas all other sensory attributes did not show statistically significant differences (*p* > 0.05).

Consequently, it can be stated that, based on the CATA analysis, enzymatic treatments with papain and bromelain did not significantly modify the sensory profile of the analyzed samples, although the selection frequencies suggest certain variation trends among the groups.

For most sensory attributes considered favorable, the enzyme-treated samples showed slightly higher selection frequencies compared with the control sample; however, these variations were not statistically confirmed for most descriptors. Thus, high selection frequencies were observed for attributes such as uniform color, characteristic pork aroma, pleasant and balanced aroma, elastic texture, juiciness, and overall acceptability.

These results suggest a slight influence of proteolytic enzyme additions, namely papain and bromelain, on sensory perception. However, the differences were not substantial, as reflected by the absence of statistically significant differences among the studied groups for most attributes (*p* > 0.05).

The enzyme-treated samples showed a relatively similar visual perception, with selection frequencies for certain visual sensory attributes being very close to those recorded for the control sample. Slightly higher selection frequencies than in the control were observed particularly for the attribute “Uniform color”; however, for other visual attributes, such as “Pinkish-red color”, “Glossy appearance in cross-section”, and “Dry surface”, the differences among samples were small and statistically non-significant (*p* > 0.05).

At the same time, the higher selection frequencies for “Fibrous appearance” and “Presence of a white layer” in the enzyme-treated samples, especially at higher enzyme concentrations, may indicate that enzymatic treatment influenced the perception of the internal structure of the product. This may represent an additional explanation, besides the more pronounced tenderization effect observed in the texture results (Table 6), for the reduction in the mean hedonic texture score (Figure 1) recorded in the samples treated with higher enzyme concentrations (LPAP-3 and LBRO-3).

Regarding olfactory and gustatory perception, the enzyme-treated samples showed selection frequencies comparable to those of the control sample (LCT-0) for attributes such as “Characteristic meat odor”, “Spice odor”, “Smoky odor”, “Characteristic pork flavor”, and “Pleasant balanced flavor”. Although slightly higher values were observed for some samples, these differences were not statistically significant (*p* > 0.05), suggesting that enzymatic additions did not markedly influence these sensory components in the analyzed products.

For all these favorable attributes, some of the highest selection frequencies were observed in the enzyme-treated groups. However, for the attribute “Overall acceptability”, the values were very close among samples (22–24), with no statistically significant differences (*p* > 0.05). This indicates that, although enzymatic treatments may have influenced certain specific sensory perceptions, the overall acceptability of the products remained relatively constant across the analyzed groups.

By correlating the results presented in Table 8 with those of the hedonic analysis (Figure 1), it can be inferred that the variations observed in CATA selection frequencies did not translate into major differences in overall acceptability. This suggests that the sensory changes induced by the enzymes were limited or not sufficiently pronounced to clearly modify consumer preference.

Nevertheless, some less desirable attributes, such as “Unusual odors”, “Sour taste”, “Bland taste”, and “Crumbly texture”, showed only minor variations in selection frequencies among samples, without statistical significance (*p* > 0.05). This indicates that enzymatic treatments did not lead to a clear intensification of these negative sensory attributes.

Consequently, Table 8 confirms that the addition of papain and bromelain generated only limited sensory differentiation in the CATA analysis, with the only statistically significant difference being observed for the attribute “Presence of a white layer”. For the other attributes, the samples showed relatively similar selection frequencies, suggesting a limited sensory effect of enzyme addition on the pork loin samples.

**Figure 2:**
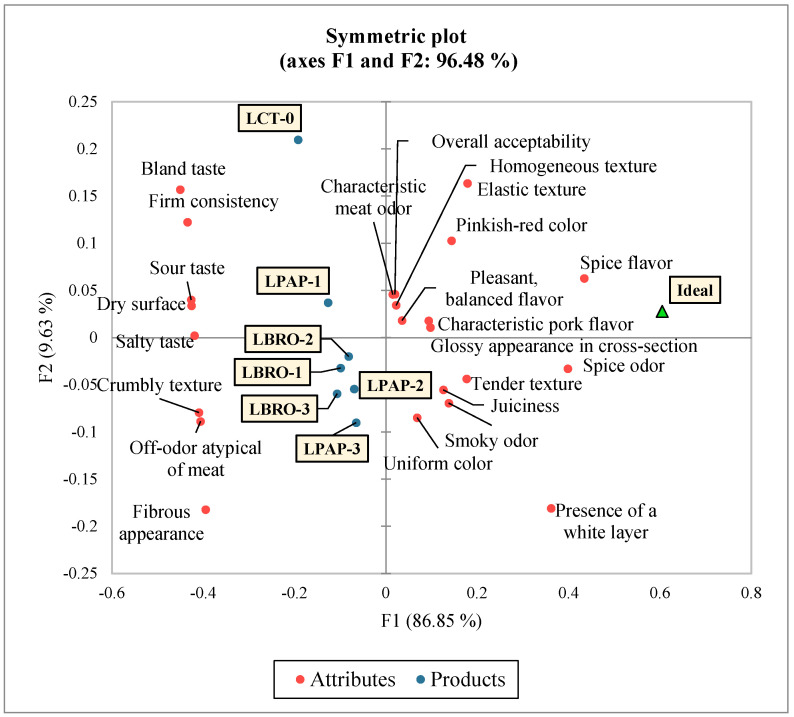
Symmetric plot of CATA sensory attributes, pork loin samples, and the ideal product profile based on the first two dimensions (F1 and F2), explaining 96.48% of the total variability. The green triangle indicates the ideal product profile. LCT-0—control sample; LPAP-1, LPAP-2, and LPAP-3—samples treated with papain at 0.015%, 0.030%, and 0.045%, respectively; LBRO-1, LBRO-2, and LBRO-3—samples treated with bromelain at 0.015%, 0.030%, and 0.045%, respectively.

Figure 2 presents the correlations among the pork loin samples, CATA sensory attributes, and the ideal profile. The first two axes of the symmetrical plot explain 96.48% of the total variability (F1 = 86.85%; F2 = 9.63%), indicating a very strong two-dimensional representation of the interactions between the samples and the sensory descriptors. Therefore, the separation observed in the factorial plot can be considered relevant for evaluating perceptual variations among the sample groups.

Overall, the F1 axis appears to represent the main dimension of sensory discrimination, separating, on the left side, the samples associated with less favorable attributes or attributes less closely related to the ideal profile, such as “Bland taste”, “Firm consistency”, “Dry surface”, “Sour taste”, “Crumbly texture”, “Odor unusual for meat”, and “Fibrous appearance”. In contrast, the right side of the axis includes samples correlated with descriptors more closely associated with consumer acceptability, namely “Overall acceptability”, “Homogeneous texture”, “Pleasant and balanced flavor”, “Juiciness”, and “Characteristic meat odor”.

Therefore, the shift in the samples toward the right side of the plot may be interpreted as a general tendency toward a more favorable sensory profile, closer to the ideal product.

The control sample, LCT-0, is clearly positioned in the upper-left quadrant, far from the enzyme-treated samples, indicating a distinct sensory profile that is less closely associated with the ideal product. Its relative association with “bland taste” and “firm consistency”, together with the descriptor “characteristic meat odor”, suggests that the control sample was perceived as a product with a traditional sensory identity, but one that was less expressive and less optimized in terms of palatability.

This positioning is consistent with the literature on untreated meat compared with enzymatically tenderized batches. Plant enzymes reduce mechanical resistance by degrading myofibrillar proteins and connective tissue components, thereby modifying the sensory profile toward increased tenderness and, under certain conditions, higher acceptability. However, this effect is dose-dependent and proportional to the intensity of proteolysis [87,88].

A distinct distribution can be observed among the papain-treated samples. LPAP-1 is positioned between the control sample and the group of samples closest to the ideal profile, indicating a stage of sensory transition. Its association with a dry surface and sour taste, together with its proximity to the control sample, suggests that the tenderizing effect was insufficient at this treatment level to fully reposition the product toward the ideal profile.

At the same time, its position closer to the center than that of LCT-0 suggests that changes in the sensory profile had already become perceptible at the 0.015% papain concentration. This situation is consistent with the mechanism of action of papain, which hydrolyzes both myofibrillar proteins and connective tissue components. However, when its action is moderate or unevenly distributed within the tissue, the sensory outcome may even become unfavorable, as reported by Bekhit et al. [28], who observed the development of a pasty and granular texture, together with a bitter taste. Under the experimental conditions of the present study, enzyme addition enhanced product tenderness without inducing any of the negative sensory effects described above.

LPAP-2 was one of the samples showing the strongest affinity with the region of positive attributes. Its positioning near the descriptors “Tender texture”, “Juiciness”, “Smoky odor”, and “Uniform color” suggests that this group benefited from a more balanced level of proteolysis, sufficient to improve tenderness and perceived juiciness without inducing evident sensory defects.

The relationship between tenderness and juiciness is particularly relevant. In meat, the perception of juiciness is influenced not only by the actual moisture content, but also by the ease with which the meat breaks down during mastication and by the release of fluids in the oral cavity. However, if proteolysis becomes excessive and cooking losses increase, this effect may be reversed [89]. In addition, papain is not commonly used in the manufacture of high-quality meat products because it may reduce juiciness and impart a bitter taste, a phenomenon associated with the formation of bitter peptides during enzymatic proteolysis [30].

These negative effects were not observed under the conditions of the present study. The sample treated with 0.045% papain, LPAP-3, was located in the lower-central region of the plot and was positioned further to the left than LPAP-2, indicating a less favorable sensory profile than LPAP-2, while still remaining distinct from the control sample. Its proximity to the attribute “Crumbly texture” indicates that the highest papain concentration was associated with a more fragile texture. This observation is consistent with literature reports showing that high papain levels can cause excessive softening, crumbly texture, and undesirable flavor notes derived from hydrolyzed peptides [28].

Gerelt et al. [90], based on their review of the literature, observed that the addition of proteolytic enzymes can enhance product tenderness; however, this effect may also be accompanied by textural deterioration, which could be described as mushy or grainy. In the present study, although the sample treated with the highest papain level (0.045%) also showed textural depreciation, the change was expressed differently, with the texture becoming crumblier.

The bromelain-treated samples also showed an interesting behavior. LBRO-1, LBRO-2, and LBRO-3 were grouped in a particularly dense area in the lower-left part of the map, suggesting that the assessors perceived them as being more similar to each other than to the papain-treated samples or to the ideal model. However, this grouping does not imply complete identity, but rather a shared sensory profile dominated by several distinctive attributes.

Their association with the descriptors “Crumbly texture”, “Odor unusual for meat”, and “Fibrous appearance” indicates that bromelain treatment had a clear influence on product structure, although not necessarily in a fully favorable way from a sensory perspective. These results are consistent with available studies, as bromelain effectively breaks down connective tissues and reduces shear force in pork meat; however, this proteolysis may affect processing quality and generate perceptible changes in texture and aroma when the treatment intensity is too high. In a study on pork loin, bromelain treatment reduced shear force and indicated endomysial hydrolysis, while the authors also noted changes in certain technological characteristics, as well as the possibility of alterations in taste and odor [34,88].

Overall, the CATA analysis indicates that the addition of proteolytic enzymes influenced the sensory profile of pork loin, although the effect was not uniform across the enzyme types and concentrations used. Papain appeared to generate a treatment intensity-dependent response, ranging from an intermediate profile (LPAP-1), to one more closely associated with positively evaluated characteristics, particularly LPAP-2, and then to a slight sensory alteration at higher doses (LPAP-3).

Although bromelain is known for its tenderizing properties, it appeared to have a stronger impact on the structural modifications in the samples, which the sensory panel described as crumbly, fibrous, and characterized by a slightly differentiated olfactory profile, namely “Off-odor atypical of meat”. These findings are consistent with the scientific literature, which shows that, although both enzymes can enhance tenderness, their broad hydrolytic action may also cause texture defects, reduced juiciness, or unpleasant notes when the dose and reaction time are not properly controlled [28,90].

**Figure 3:**
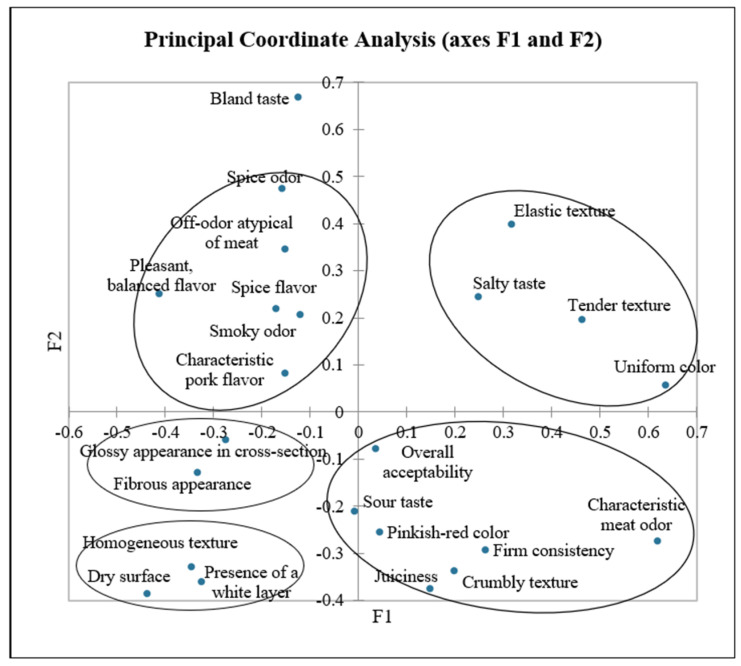
Representation of the relationships among CATA sensory attributes in the principal coordinate analysis plane (F1 and F2), based on their concurrent selection by the evaluators. LCT-0—control sample; LPAP-1, LPAP-2, and LPAP-3—samples treated with papain at 0.015%, 0.030%, and 0.045%, respectively; LBRO-1, LBRO-2, and LBRO-3—samples treated with bromelain at 0.015%, 0.030%, and 0.045%, respectively.

The distribution of the CATA sensory attributes within the space defined by the F1 and F2 axes is illustrated in Figure 3, allowing the relationships among descriptors to be observed according to how the assessors associated them during the evaluation of pork loin samples treated with proteolytic enzymes. Notably, the attributes formed several distinct association areas rather than being randomly distributed, indicating the presence of well-defined sensory patterns.

The sensory attributes located in the upper-left quadrant are represented by “Bland taste”, “Spice odor”, “Off-odor atypical of meat”, “Pleasant and balanced flavor”, “Smoky odor”, and “Characteristic pork flavor”. Spicy and smoky aromatic and gustatory impressions were associated with this group; however, the simultaneous presence of the descriptor “Off-odor atypical of meat” suggests that this area does not entirely represent a favorable profile. In other words, the panelists reported a mixed sensory core in this region, in which the characteristic pork odor and spicy notes coexisted with a slight deviation from the conventional sensory profile. The results of the present study indicate that the alteration in the sensory profile through the use of proteolytic enzymes was strictly dose-dependent. This inconsistency with the typical attributes of the product, caused by the excessive intensification of certain aromatic notes, was identified exclusively at the highest enzyme addition levels for both enzyme types used, namely papain and bromelain.

A second group, which includes “Elastic texture”, “Tender texture”, “Salty taste”, and “Uniform color”, highlights a strong correlation between pleasant visual appearance and favorable textural qualities. The association between elasticity and tenderness is particularly important, as it shows that the desired texture was determined by a combination of structural resilience and ease of mastication, rather than by a single textural dimension.

The idea of an overall harmonious profile is further emphasized by the inclusion of the sensory attribute “Uniform color” within this group, where a pleasant appearance is combined with appropriate texture and, tangentially, with the taste characteristics of the samples.

“Juiciness”, “Firm consistency”, “Crumbly texture”, “Pinkish-red color”, “Characteristic meat odor”, “Sour taste”, and “Overall acceptability” constitute another important category. The greater heterogeneity of this group suggests a more complex sensory experience. On the one hand, attributes such as “Juiciness”, “Pinkish-red color”, and “Characteristic meat odor” may reinforce a favorable impression, as they are often associated with freshness and the sensory quality of meat products. However, the inclusion of “Sour taste” and “Crumbly texture” indicates that this profile also contains some potentially limiting aspects. For this reason, the positioning of “Overall acceptability” within this group suggests that general acceptance was determined by the extent to which appreciated qualities, such as juiciness, color, and characteristic meat odor, compensated for possible negative attributes. Therefore, rather than representing a clearly positive or strictly negative profile, this group reflects the complexity of a sensory trade-off.

On the opposite side of the plot from the group discussed above, two groups can be observed, characterized by “Dry surface”, “Homogeneous texture”, “Presence of a white layer”, “Fibrous appearance”, and “Glossy appearance in cross-section”. Unlike the previous groups, these clusters are dominated more by structural and visual characteristics than by olfactory or gustatory perceptions. The association between fibrous appearance and dry surface indicates a profile perceived as less attractive and less juicy.

In essence, Figure 3 illustrates how the CATA descriptors were grouped into a series of coherent sensory clusters. As a result, this study highlights the multifaceted nature of the sensory profile, showing not only which attributes were selected, but especially how the assessors formed their perception of the product through recurring associations among specific sensory traits.

#### 3.5.3. Quantitative Descriptive Analysis

Quantitative descriptive analysis showed that, although taste and olfactory attributes remained largely unchanged, the addition of proteolytic enzymes mainly affected appearance and texture (Table 9). Overall, the results indicate that enzyme addition led to structural changes in the analyzed samples without negatively affecting their sensory identity. This had a direct impact on the perception of tenderness, elasticity, firmness, and juiciness, for which significant differences were identified (*p* < 0.05).

These results are consistent with the scientific literature, which demonstrates that papain and bromelain act by hydrolyzing peptide bonds, breaking down proteins from muscle and connective tissues into peptides and, ultimately, amino acids, thereby reducing meat toughness [30].

One of the most evident visual changes was observed for the attribute “Color uniformity”, for which all enzyme-treated samples obtained higher scores than the control sample (6.53 ± 0.18). The highest mean scores were recorded for LPAP-3 and LBRO-3, with values of 7.70 ± 0.16 and 7.73 ± 0.17, respectively. The effect of enzyme concentration was statistically extremely significant (*p* < 0.0001). On the other hand, the attribute “Pinkish-red color” showed a slight decrease as treatment intensity increased, particularly in the case of papain, where LPAP-3 decreased to 5.30 ± 0.27 compared with 6.07 ± 0.21 in the control sample (LCT-0). The reduction in pinkish-red color was slightly more evident in the papain-treated groups than in the bromelain-treated groups. However, the changes observed for this sensory parameter were not statistically significant either in relation to enzyme type or enzyme concentration (*p* > 0.05).

With a highly significant effect of enzyme concentration (*p* < 0.0001), regardless of enzyme type (*p* > 0.05), the fibrous appearance of the pork loin samples recorded higher mean scores, increasing from 5.67 ± 0.19 in the control sample to a range of 6.77–6.97 in all enzyme-treated samples. Similarly, the presence of a white layer increased from 2.53 in the control sample to approximately 3.63–4.17 in the treated samples. These changes were common to both enzyme types, with no significant differences depending on the enzyme used (*p* > 0.05). However, enzyme concentration was the determining factor for the mean score of this parameter, leading to extremely significant statistical differences (*p* < 0.001). The literature indicates that this whitish layer perceived by the assessors may be caused by the addition of proteolytic enzymes, which promotes the release of exudate rich in solubilized protein fractions. Similar findings in beef were reported by Ionescu et al. [91]. According to the study by Sheard et al. [92], the white exudate observed on the meat surface contains proteins and consists mainly of sarcoplasmic proteins, namely solubilized proteins. Therefore, the whitish appearance observed in the present study may be explained by the migration of a soluble protein fraction to the surface, becoming visible after thermal treatment.

Regarding the odor and taste of the pork loin samples, the effects induced by papain and bromelain addition were generally limited and statistically non-significant (*p* > 0.05), except for “Smoky odor”, for which extremely significant differences (*p* < 0.0001) were obtained in relation to enzyme concentration, but not enzyme type. All enzymatically treated samples received higher scores for this sensory attribute, with mean values ranging from 6.17 to 6.50, compared with the control sample, which recorded a mean score of 5.33. This suggests that enzymatic treatment enhanced the olfactory perception of smoking. At the same time, the addition of papain and bromelain did not intensify negative olfactory perceptions, such as “Foreign odor, atypical of meat”. Although the mean values of the enzyme-treated samples were slightly higher than those of the control sample, the differences were not statistically significant (*p* > 0.05), suggesting that these perceptions were only very subtle and did not have a relevant sensory impact.

From a gustatory perspective, the differences were statistically non-significant (*p* > 0.05). Salty, sour, and bland taste perceptions were not significantly affected, while characteristic pork flavor and pleasant, balanced flavor remained at levels comparable to those of the control sample, even recording slightly higher mean scores. Consequently, enzymatic treatments had only a limited effect on the taste profile, although a noticeable enhancement in smoky odor was observed at the olfactory level.

At the sensory level, texture-related attributes showed the most relevant statistical differences. “Elastic texture” (*p* = 0.001), “Tender texture” (*p* = 0.002), “Firmness” (*p* = 0.008), and “Juiciness” (*p* = 0.002) were significantly modified depending on enzyme concentration (F2). However, since the *p*-values were consistently higher than 0.05, enzyme type (F1) did not have a significant effect on any of these descriptors. Based on these results, it can be inferred that the textural effects were similar for both papain and bromelain.

Scores for “Elastic texture” decreased from 5.60 ± 0.14 in the control sample (LCT-0) to 4.80 ± 0.18 in the sample treated with the highest papain concentration (LPAP-3) and to 4.70 ± 0.19 in the sample treated with the highest bromelain concentration (LBRO-3), suggesting that increasing enzyme concentration led to a decrease in overall elasticity. Firmness values showed a similar pattern, decreasing from 5.53 ± 0.15 in the control sample (LCT-0) to 4.90 ± 0.16 in LPAP-3 and 4.70 ± 0.19 in LBRO-3, indicating that proteolytic treatment weakened the product structure.

At the same time, tenderness scores increased from 5.07 ± 0.17 in the control sample (LCT-0) to 5.80 in the variants treated with papain at doses of 0.030% and 0.045%, and to 5.57 ± 0.14 in the sample treated with the highest bromelain concentration. This demonstrates an increase in perceived tenderness as the amount of added enzyme increased. For tenderness, enzyme type showed a differentiated effect, with higher tenderness scores being obtained in the bromelain-treated samples compared with the papain-treated ones. However, these differences were not statistically significant (*p* > 0.05). Mean scores for the juiciness attribute also increased in the enzyme-treated samples, from 5.77 ± 0.24 in LCT-0 to 6.57 ± 0.20 in LPAP-3 and 6.67 ± 0.18 in LBRO-3, suggesting that the treated samples were perceived as juicier than the control sample.

“Homogeneous texture” (F1: *p* = 0.242; F2: *p* = 0.987), “Crumbly texture” (F1: *p* = 0.386; F2: *p* = 0.879), and “Overall acceptability” (F1: *p* = 0.747; F2: *p* = 0.429) did not show statistically significant differences (*p* > 0.05). These results suggest that the changes induced by the addition of papain and bromelain did not negatively affect the overall perception of the pork loin samples.

Juiciness is considered a major sensory parameter in the assessment of meat product quality, contributing significantly to the overall acceptability of meat products, together with tenderness and flavor [93,94]. Compared with the control sample, treatments with papain and bromelain increased juiciness scores. This result is consistent with those reported by Bhattarai and Lamichhane [86], who highlighted an improvement in sensory quality following proteolytic treatment with both enzymes. In the present study, the increase in juiciness was very significantly influenced by enzyme concentration (*p* = 0.002), whereas the absence of significant differences between papain and bromelain (*p* = 0.429) indicates that the two enzymes had comparable effects on this descriptor.

#### 3.5.4. Principal Component Analysis (PCA)

**Figure 4:**
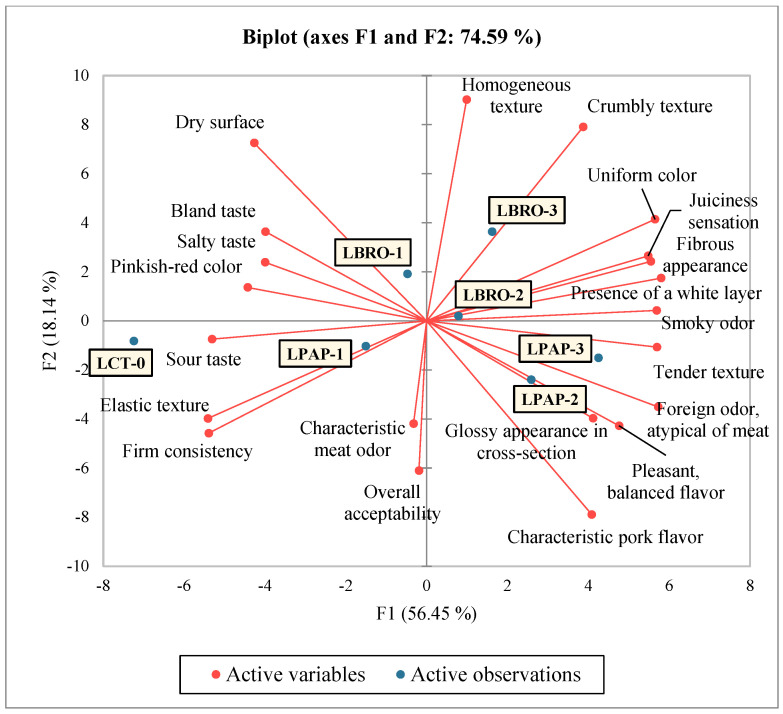
Principal component analysis (PCA) biplot of the sensory attributes and sample distribution. LCT-0—control sample; LPAP-1, LPAP-2, and LPAP-3—samples treated with papain at 0.015%, 0.030%, and 0.045%, respectively; LBRO-1, LBRO-2, and LBRO-3—samples treated with bromelain at 0.015%, 0.030%, and 0.045%, respectively.

Figure 4, represented by a PCA biplot, highlights the relationships between the sensory descriptors and the analyzed samples, with interpretation being based primarily on the orientation of the vectors and the relative position of the samples with respect to them. The angle between two vectors is used to determine the correlation between the corresponding attributes: an acute angle indicates a positive correlation, an obtuse angle indicates a negative correlation, and a right angle indicates the absence of correlation [95].

“Juiciness”, “Fibrous appearance”, “Presence of a white layer”, “Smoky odor”, “Tender texture”, “Pleasant and balanced flavor”, and “Characteristic pork flavor” were among the sensory attributes correlated on the right side of the biplot. The relatively small angles between these vectors indicate a positive correlation among these characteristics. Consequently, samples perceived as juicier also tended to exhibit a more pronounced fibrous appearance, a more tender texture, and a more pleasant or more clearly defined olfactory profile. The proximity of sample LBRO-2 to these descriptors indicates that this sample was most strongly characterized by them.

In contrast, the sample treated with the highest bromelain concentration in the present study (LBRO-3, 0.045% bromelain) shared, to some extent, the characteristics of the sample treated with 0.030% bromelain (LBRO-2). However, the increase in bromelain concentration also led to the development of a crumbly texture. This was likely one of the reasons for the decrease in the overall mean scores for appearance and texture recorded for this sample in the hedonic analysis (Figure 1), even though its texture appeared to be more homogeneous in the PCA biplot (Figure 4).

The proximity between samples LPAP-2 and LPAP-3 suggests that their sensory profiles were similar. These samples were mainly associated with sensory attributes such as “Glossy appearance in cross-section”, “Tender texture”, “Pleasant and balanced flavor”, “Characteristic pork flavor”, and “Foreign odor atypical of meat”. In contrast, sample LPAP-1 was positioned farther away from the other papain-treated samples and was more strongly correlated with sensory characteristics such as “Sour taste”, “Characteristic meat odor”, and “Overall acceptability”. The latter two attributes also showed a slight association with the other two papain-treated samples (LPAP-2 and LPAP-3), as these were located at an intermediate distance relative to LPAP-1 and the corresponding sensory descriptors.

Sample LCT-0 was slightly distinguished from the other samples, as it was positioned on the opposite side of the plot from the sensory attributes related to “Juiciness”, “Tender texture”, “Pleasant taste”, and “Characteristic pork flavor”, which were more closely associated with samples LPAP-2, LPAP-3, LBRO-2, and LBRO-3. This positioning indicates a lack of correlation between LCT-0 and these attributes. Instead, LCT-0 was associated with sensory attributes such as “Firm consistency”, “Sour flavor”, “Elastic texture”, and a “Pinkish-red color”. This suggests that the control sample was perceived as being less associated with juiciness and tenderness attributes, while being firmer and more elastic.

The control sample (LCT-0) was positioned very close to LPAP-1, indicating that these two samples shared broadly similar sensory characteristics. This positioning suggests that a low papain concentration (0.015%) did not markedly influence the sensory profile of pork loin, with more clearly perceptible differences being identified at concentrations of 0.030% and 0.045%.

Sample LBRO-1 was also located relatively close to the control sample (LCT-0), but was oriented more toward sensory attributes such as “Bland taste”, “Salty taste”, “Pinkish-red color”, and “Dry surface”. This suggests that LBRO-1, treated with 0.015% bromelain, retained some sensory characteristics close to those of the control sample, although to a lesser extent than the sample treated with 0.015% papain (LPAP-1). These results indicate that, at lower concentrations, bromelain induced a slightly more pronounced sensory modification in pork loin than papain. However, at higher concentrations, the sensory changes became more similar between the two enzymes.

Regarding the correlations among sensory attributes, “Firm consistency” and “Elastic texture” were closely correlated with each other, but positioned in opposition to “Tender texture”, “Juiciness”, and “Pleasant taste”, forming an angle greater than 90°. This indicates that firmer and more elastic samples were perceived as less juicy and less tender. In addition, a positive correlation was observed among “Dry surface”, “Bland taste”, “Salty taste”, and “Pinkish-red color”. These attributes were negatively correlated with favorable sensory characteristics such as “Juiciness”, “Tenderness”, “Smoky odor”, and “Pleasant, balanced flavor”.

#### 3.5.5. Preference Mapping

**Figure 5:**
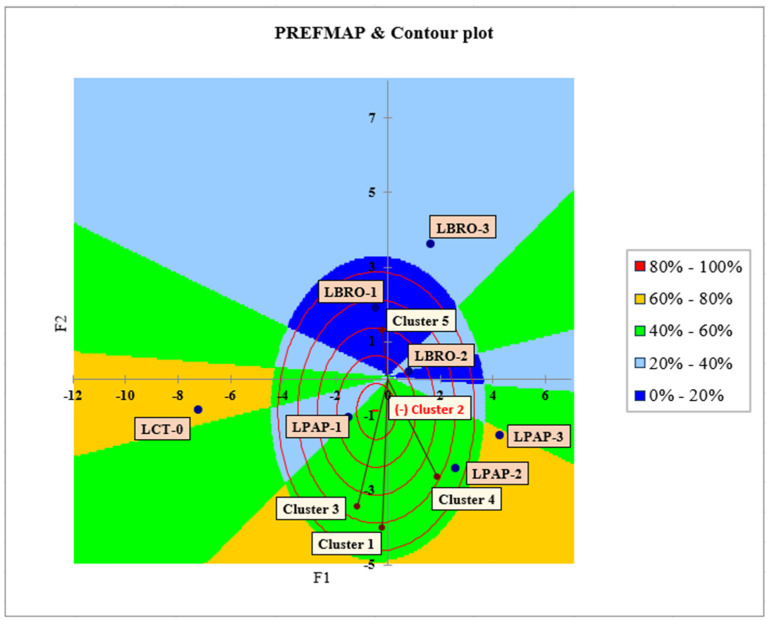
Preference map and contour plot of pork loin samples treated with papain and bromelain, showing consumer preference levels and sensory cluster distribution. LCT-0—control sample; LPAP-1, LPAP-2, and LPAP-3—samples treated with papain at 0.015%, 0.030%, and 0.045%, respectively; LBRO-1, LBRO-2, and LBRO-3—samples treated with bromelain at 0.015%, 0.030%, and 0.045%, respectively.

The preference map shown in Figure 5 illustrates the distribution of samples according to consumer preferences, combining the hedonic perception of the samples with the quantitative descriptive characteristics obtained from the sensory analysis of these samples [96]. The results of this statistical analysis indicate the optimal preference region through the red concentric circles shown in the figure. In this type of representation, samples located at or as close as possible to the center of these circles correspond to the sensory profile perceived by consumers as ideal, whereas samples positioned farther from the center show a lower similarity to the optimal profile established by consumers.

Among the analyzed pork loin samples, LPAP-1 and LBRO-2 were positioned closest to the center of the preference circles. These samples showed the best correspondence with the sensory profile considered ideal by the assessors. Samples treated with 0.015% bromelain (LBRO-1) and 0.030% papain (LPAP-2) were also located within the consumer preference area, although slightly farther from the center of ideal preference. The remaining analyzed samples, namely LBRO-3, LPAP-3, and LCT-0, were positioned in different areas of the preference map outside the central preference region, indicating that they matched the main pattern of consumer preferences to a lesser extent.

The spatial distribution of the samples on the preference map highlights that the addition of proteolytic enzymes had variable effects on consumer perception depending on both the type of enzyme added and its concentration. The diverse distribution of the samples on the PrefMap, regardless of enzyme type, suggests that enzyme type was not the main factor responsible for sensory differences and sample preference; rather, this role was mainly attributed to enzyme concentration.

The positioning of the samples treated with 0.015% enzyme addition (LPAP-1 and LBRO-1) and 0.030% enzyme addition (LPAP-2 and LBRO-2) within the concentric circles indicates that lower enzyme concentrations led to a sensory balance between hedonic perception and descriptive sensory attributes that was closer to consumer preferences. This result was observed together with an improvement in the sensory quality of texture without causing excessive softening of the muscle structure. This interpretation is supported by the fact that the quantitative descriptive textural attributes showed the most significant differences among samples, being the attributes most strongly influenced by enzyme addition and enzyme concentration, as shown in Table 9.

Increasing the concentration of proteolytic enzymes to 0.045% (LPAP-3 and LBRO-3) led to a reduction in consumer preference for these samples. Similar results were also observed in the hedonic analysis (Figure 1), where increasing enzyme addition to 0.045% resulted in a decrease in consumer hedonic perception, with this reduction being more evident for the bromelain-treated sample (LBRO-3) than for the papain-treated sample (LPAP-3). Similar trends to those observed in the hedonic analysis can also be identified in the preference map. Both LPAP-3 and LBRO-3 were located outside the central preference circles. However, LPAP-3 remained within the 60–80% consumer preference area, whereas LBRO-3 was positioned in the lower 20–40% preference range.

Although the control sample (LCT-0) was not located within the ideal preference circle, it was positioned in the 60–80% consumer preference range, indicating that it was well accepted by the assessors, as also observed in the hedonic analysis (Figure 1). However, its descriptive sensory characteristics were inferior to those of the samples treated with 0.015% and 0.030% enzyme addition (Table 9), which explains its positioning outside the ideal consumer preference area.

The positioning of consumer clusters within the preference map also highlights a slight variability in consumer preferences. However, it can be observed that these clusters are located only within the ideal preference circles. Cluster 2, positioned exactly at the center of the preference circles, indicates that the sensory responses of this consumer group correspond to the optimal profile identified by this sensory analysis, showing preferences oriented toward the combination of hedonic and quantitative descriptive sensory characteristics that define the maximum preference area of the preference map.

In conclusion, the results of the sensory evaluation based on the external preference map show that pork loin samples treated with low or moderate concentrations of proteolytic enzymes (LPAP-1 and LBRO-2) showed the best correspondence with the sensory profiles preferred by consumers. In contrast, samples with higher enzyme concentrations were less appreciated, demonstrating that increasing enzyme concentration reduced the assessors’ level of satisfaction with the product.

Furthermore, papain-treated samples showed a slightly higher preference level than bromelain-treated samples. However, the distribution of the samples on the preference map indicates that enzyme concentration had the greatest impact on the assessors’ response.

## 4. Conclusions

This study demonstrated that the incorporation of papain and bromelain into a red algae-based brine containing *Palmaria palmata* is an effective approach for modifying the quality attributes of injected pork loin, particularly texture and sensory perception. The enzymatic treatments induced only minor changes in the proximate composition of the samples, indicating that the addition of these plant proteases did not substantially alter the basic chemical profile of the product. Moisture and dry matter varied within a narrow range, protein content remained largely stable, and the main compositional changes consisted of a reduction in fat content and energy value, especially at higher enzyme concentrations.

The water activity and pH results further support the observation that papain and bromelain did not substantially modify the chemical composition of the pork loin samples. Water activity remained stable across all treatments, indicating that the enzymes did not affect the overall availability of free water in the product. However, pH was significantly influenced, mainly by enzyme type, with higher values observed in bromelain-treated samples. These findings suggest that the main effect of the enzymatic treatments was related primarily to structural modifications in muscle proteins, with direct implications for texture and tenderness, rather than to major changes in chemical composition or water stability.

The enzymatic treatments also had a significant effect on the instrumental color of the pork loin samples. In general, treated samples showed increased lightness and reduced redness and chroma compared with the control, while Δ*E* values indicated visible color differences among formulations. These findings indicate that papain and bromelain influenced the optical properties of the product. Since direct structural protein degradation was not assessed, the color changes should be interpreted as treatment-dependent effects rather than as confirmed enzyme-induced structural modifications. From a technological perspective, this finding is important because the tenderizing benefits of plant proteases must be balanced against their potential impact on visual quality. Therefore, enzyme dosage should be carefully optimized to improve texture while avoiding excessive changes in redness, color saturation, and overall appearance.

The most pronounced effect of the enzymatic treatments was observed in the textural profile. Both papain and bromelain significantly reduced Warner–Bratzler shear force, work of shear, hardness, gumminess, chewiness, springiness, and adhesiveness, confirming the tenderizing effect of these plant proteases. Enzyme concentration had a stronger influence than enzyme type on most instrumental texture parameters, indicating that the intensity of proteolysis was the main factor responsible for the weakening of the muscle structure. The highest enzyme concentrations produced the most marked textural changes, with LBRO-3 showing the lowest shear force and LPAP-3 showing strong reductions in hardness, gumminess, and chewiness. However, the sensory results indicate that excessive enzymatic treatment may lead to less desirable textural perceptions, such as crumbliness, particularly in the sample treated with the highest bromelain concentration.

The integrated sensory assessment showed that the samples treated with 0.030% enzyme addition, namely LPAP-2 and LBRO-2, achieved the highest appreciation and the most balanced sensory profile. These formulations combined improved tenderness and juiciness with pleasant flavor, smoky notes, characteristic pork flavor, and good overall acceptability. This suggests that an intermediate level of proteolytic treatment provided a better sensory compromise than the highest enzyme concentrations, which, although effective in reducing instrumental firmness, were associated with less desirable textural perceptions such as crumbliness. Thus, the sensory data indicate that maximum consumer acceptability depends not only on increasing tenderness, but also on maintaining a balanced relationship between tenderness, juiciness, flavor, and structural integrity.

Taken together, the results indicate that papain and bromelain can be successfully used as natural tenderizing agents in injected pork loin when incorporated into a *Palmaria palmata*-based brine. The formulation containing *Palmaria palmata* was associated with stable water-related parameters, while papain and bromelain improved tenderness-related characteristics. However, the specific contribution of *Palmaria palmata* to water balance should be confirmed in future studies using formulations without algae as additional controls. This combined approach allowed for the development of pork loin samples with improved textural properties, enhanced juiciness, and higher sensory scores. Among the tested variants, the intermediate enzyme treatments (0.030%) provided the best balance between tenderization, moisture-related sensory perception, flavor, and structural integrity. These findings support the use of plant proteases in red algae-based formulations for developing whole-muscle pork products with improved eating quality and preserved microbiological suitability under the conditions tested in this study. Future studies should evaluate the degree of proteolysis, free amino acid formation, peptide profile, oxidative stability, water-holding capacity, cooking loss, and longer storage periods in order to better understand the mechanisms underlying the quality changes induced by papain and bromelain in *Palmaria palmata*-formulated meat systems.

## Figures and Tables

**Table 1:** Composition of the brine for the experimental batches (%).

Ingredients	LCT-0	LPAP-1	LPAP-2	LPAP-3	LBRO-1	LBRO-2	LBRO-3
Water	79.00	78.835	78.670	78.505	78.835	78.670	78.505
Salt	10.00	10.00	10.00	10.00	10.00	10.00	10.00
Papain	0.00	0.165	0.330	0.495	0.000	0.000	0.000
Bromelain	0.000	0.000	0.000	0.000	0.165	0.330	0.495
Algae	11.00	11.00	11.00	11.00	11.00	11.00	11.00

**Table 2:** Thermal treatment stages and processing parameters.

Technological Stage	Time (min.)	Temperature (°C)	Core Temperature of the Product (°C)	Humidity (%)
First drying phase	30	62	58	10
Smoking	40	68	62	10
Thermal treatment	120	78	72	99
Second drying phase	10	76	50	10

**Table 3:** Sensory attributes and terms used for sample evaluation.

Sensory Attributes	Sensory Terms Used
Appearance	uniform color, rosy-red color, pinkish-white color, shiny appearance in cross-section, fibrous appearance, presence of a white layer, dry surface
Odor	specific meat odor, smoke odor (smoked), foreign odor (non-specific to meat), spice odor
Taste	specific pork taste, pleasant, balanced taste, salty taste, sour taste, bland taste, spice flavor
Texture	elastic texture, tender texture, homogeneous texture, crumbly texture, hard consistency, sensation of juiciness (succulence)
Overall Evaluation	overall acceptability

**Table 4:** Chemical composition of control and enzyme-treated samples and the significance of enzyme type and concentration effects.

Chemical Parameters	Control	Papain-Treated Samples			Bromelain-Treated Samples			Significance of Effects (*p*-Value)	
	LCT-0	LPAP-1	LPAP-2	LPAP-3	LBRO-1	LBRO-2	LBRO-3	Enzyme Type	Enzyme Concentration
Dry matter, %	25.84 ± 0.16 ^b^	25.68 ± 0.09 ^ab^	25.63 ± 0.07 ^a^	25.56 ± 0.08 ^a^	25.72 ± 0.05 ^ab^	25.69 ± 0.05 ^ab^	25.67 ± 0.04 ^ab^	0.039 *	0.102 ^ns^
Moisture, %	74.16 ± 0.16 ^a^	74.32 ± 0.09 ^ab^	74.37 ± 0.07 ^b^	74.44 ± 0.08 ^b^	74.28 ± 0.05 ^ab^	74.31 ± 0.05 ^ab^	74.33 ± 0.04 ^ab^		
Fat, %	3.09 ± 0.09 ^c^	2.88 ± 0.06 ^b^	2.80 ± 0.09 ^ab^	2.70 ± 0.09 ^ab^	2.91 ± 0.08 ^b^	2.84 ± 0.08 ^ab^	2.82 ± 0.06 ^ab^	0.064 ^ns^	0.003 **
Protein, %	21.32 ± 0.09 ^b^	21.06 ± 0.04 ^a^	21.03 ± 0.06 ^a^	21.01 ± 0.09 ^a^	21.04 ± 0.06 ^a^	21.03 ± 0.09 ^a^	21.01 ± 0.07 ^a^	0.848 ^ns^	0.448 ^ns^
Ash, %	1.40 ± 0.05 ^a^	1.51 ± 0.04 ^b^	1.53 ± 0.05 ^b^	1.56 ± 0.07 ^b^	1.53 ± 0.04 ^b^	1.54 ± 0.03 ^b^	1.56 ± 0.01 ^b^	0.538 ^ns^	0.126 ^ns^
Carbohydrates, %	0.03 ± 0.04 ^a^	0.24 ± 0.02 ^b^	0.28 ± 0.15 ^b^	0.29 ± 0.10 ^b^	0.25 ± 0.05 ^b^	0.27 ± 0.09 ^b^	0.29 ± 0.09 ^b^	0.831 ^ns^	0.369 ^ns^
Energy value, kcal/100g	113.21 ± 1.21 ^c^	111.07 ± 0.57 ^b^	110.43 ± 0.36 ^ab^	109.49 ± 0.58 ^a^	111.32 ± 0.62 ^ab^	110.76 ± 0.42 ^ab^	110.54 ± 0.42 ^ab^	0.043 *	0.002 **
Energy value, kJ/100 g	477.28 ± 5.02 ^c^	468.44 ± 2.33 ^b^	465.81 ± 1.41 ^ab^	461.97 ± 2.35 ^a^	469.48 ± 2.54 ^b^	467.17 ± 1.69 ^ab^	466.26 ± 1.71 ^ab^		
Water activity	0.97 ± 0.02 ^a^	0.98 ± 0.01 ^a^	0.97 ± 0.01 ^a^	0.98 ± 0.01 ^a^	0.98 ± 0.00 ^a^	0.97 ± 0.00 ^a^	0.98 ± 0.00 ^a^	0.861 ^ns^	0.140 ^ns^
pH	6.04 ± 0.03 ^a^	6.02 ± 0.02 ^a^	6.03 ± 0.01 ^a^	6.04 ± 0.03 ^a^	6.06 ± 0.02 ^ab^	6.14 ± 0.01 ^c^	6.13 ± 0.08 ^bc^	<0.0001 ***	0.012 *

Different superscript letters within the same row indicate significant differences among all experimental samples, including the control and enzyme-treated samples, according to the post hoc test (*p* < 0.05). ns, not significant; * *p* < 0.05; ** *p* < 0.01; *** *p* < 0.001. LCT-0—control sample; LPAP-1, LPAP-2, and LPAP-3—samples treated with papain at 0.015%, 0.030%, and 0.045%, respectively; LBRO-1, LBRO-2, and LBRO-3—samples treated with bromelain at 0.015%, 0.030%, and 0.045%,

**Table 5:** Changes in color parameters (L*, a*, b*, C*, h*, and Δ*E*) of samples treated with papain and bromelain, depending on enzyme type and concentration.

Chemical Parameters	Control	Papain-Treated Samples			Bromelain-Treated Samples			Effect Significance (*p*-Value)	
	LCT-0	LPAP-1	LPAP-2	LPAP-3	LBRO-1	LBRO-2	LBRO-3	Enzyme Type	Enzyme Concentration
L*	68.67 ± 0.62 ^a^	73.48 ± 0.95 ^d^	74.25 ± 0.31 ^d^	71.13 ± 0.67 ^bc^	69.71 ± 0.95 ^ab^	71.65 ± 1.03 ^c^	74.25 ± 0.96 ^d^	0.001 ***	0.002 **
a*	13.07 ± 0.39 ^d^	6.46 ± 0.38 ^b^	8.13 ± 0.15 ^c^	5.19 ± 0.32 ^a^	8.15 ± 0.35 ^c^	8.45 ± 0.54 ^c^	6.66 ± 0.29 ^b^	<0.0001 ***	<0.0001 ***
b*	8.28 ± 0.27 ^a^	8.99 ± 0.40 ^cd^	9.44 ± 0.08 ^de^	8.76 ± 0.29 ^bc^	8.49 ± 0.12 ^ab^	9.39 ± 0.20 ^de^	9.63 ± 0.06 ^e^	0.219 ^ns^	<0.0001 ***
C*	15.47 ± 0.28 ^e^	11.08 ± 0.16 ^b^	12.46 ± 0.11 ^d^	10.19 ± 0.26 ^a^	11.77 ± 0.17 ^c^	12.64 ± 0.35 ^d^	11.71 ± 0.18 ^c^	<0.0001 ***	<0.0001 ***
h*	0.56 ± 0.02 ^a^	0.95 ± 0.05 ^c^	0.86 ± 0.01 ^b^	1.04 ± 0.03 ^d^	0.81 ± 0.03 ^b^	0.84 ± 0.04 ^b^	0.97 ± 0.02 ^c^	<0.0001 ***	<0.0001 ***
Δ*E*	-	8.31 ± 0.55 ^b^	7.57 ± 0.54 ^b^	8.29 ± 0.63 ^b^	5.13 ± 0.6 ^a^	5.72 ± 0.87 ^a^	8.66 ± 0.35 ^b^	<0.0001 ***	<0.0001 ***

Different superscript letters within the same row indicate significant differences among all experimental samples according to the post hoc test (*p* < 0.05). The effects of enzyme type and enzyme concentration are reported as *p*-values. ns, not significant; ** *p* ≤ 0.01; *** *p* ≤ 0.001. LCT-0—control sample; LPAP-1, LPAP-2, and LPAP-3—samples treated with papain at 0.015%, 0.030%, and 0.045%, respectively; LBRO-1, LBRO-2, and LBRO-3—samples treated with bromelain at 0.015%, 0.030%, and 0.045%, respectively. Δ*E* was calculated relative to the control sample.

**Table 6:** Texture profile parameters, Warner–Bratzler shear force, and work of shear of control, papain-treated, and bromelain-treated samples.

Parameters	Control	Papain-Treated Samples			Bromelain-Treated Samples			Effect Significance (*p*-Value)	
	LCT-0	LPAP-1	LPAP-2	LPAP-3	LBRO-1	LBRO-2	LBRO-3	Enzyme Type	Enzyme Concentration
Warner–Bratzler shear force, N/cm^2^	26.22 ± 9.21 ^b^	16.15 ± 1.68 ^a^	10.65 ± 2.26 ^a^	10.78 ± 2.28 ^a^	13.55 ± 1.74 ^a^	13.33 ± 0.57 ^a^	9.38 ± 1.81 ^a^	0.758 ^ns^	0.033 *
Work of shear, mJ	602.50 ± 145.98 ^b^	352.95 ± 30.43 ^a^	253.12 ± 53.93 ^a^	210.71 ± 47.64 ^a^	299.08 ± 52.56 ^a^	245.02 ± 25.37 ^a^	303.38 ± 76.62 ^a^	0.702 ^ns^	0.047 *
Hardness, N	41.29 ± 12.59 ^c^	28.67 ± 1.79 ^b^	16.42 ± 3.67 ^a^	15.35 ± 3.72 ^a^	20.76 ± 4.11 ^ab^	18.86 ± 1.46 ^ab^	18.49 ± 4.38 ^ab^	0.712 ^ns^	0.008 **
Cohesiveness, -	0.27 ± 0.16 ^a^	0.11 ± 0.04 ^a^	0.15 ± 0.04 ^a^	0.09 ± 0.03 ^a^	0.16 ± 0.11 ^a^	0.16 ± 0.15 ^a^	0.23 ± 0.17 ^a^	0.843 ^ns^	0.509 ^ns^
Springiness, -	0.26 ± 0.09 ^b^	0.16 ± 0.02 ^a^	0.11 ± 0.02 ^a^	0.11 ± 0.02 ^a^	0.14 ± 0.02 ^a^	0.13 ± 0.01 ^a^	0.09 ± 0.02 ^a^	0.758 ^ns^	0.033 *
Gumminess, N	7.66 ± 4.36 ^b^	3.54 ± 0.55 ^ab^	2.49 ± 1.05 ^a^	1.58 ± 0.39 ^a^	4.28 ± 3.48 ^ab^	3.02 ± 1.45 ^ab^	2.99 ± 2.67 ^ab^	0.594 ^ns^	0.044 *
Chewiness N	1.22 ± 0.71 ^c^	0.92 ± 0.31 ^bc^	0.28 ± 0.15 ^ab^	0.17 ± 0.06 ^a^	0.40 ± 0.15 ^ab^	0.40 ± 0.37 ^ab^	0.42 ± 0.36 ^ab^	0.258	0.006 **
Adhesiveness, mJ	0.67 ± 0.01 ^a^	0.64 ± 0.01 ^b^	0.65 ± 0.01 ^b^	0.64 ± 0.01 ^b^	0.62 ± 0.02 ^b^	0.60 ± 0.02 ^c^	0.60 ± 0.01 ^c^	<0.0001 ***	0.014 *

Different superscript letters within the same row indicate significant differences among all experimental samples according to the post hoc test (*p* < 0.05). The effects of enzyme type and enzyme concentration are reported as *p*-values. ns, not significant; * *p* ≤ 0.05; ** *p* ≤ 0.01; *** *p* ≤ 0.001. LCT-0—control sample; LPAP-1, LPAP-2, and LPAP-3—samples treated with papain at 0.015%, 0.030%, and 0.045%, respectively; LBRO-1, LBRO-2, and LBRO-3—samples treated with bromelain at 0.015%, 0.030%, and 0.045%, respectively.

**Table 7:** Effect of papain and bromelain treatment on the microbiological quality of samples during refrigerated storage.

Samples		TVC (×10^2^ CFU/g)			*Escherichia coli* (cfu/g) ×10^1^			*Salmonella* spp. in 10 g			*Listeria monocytogenes* in 25 g		
		D0	D7	D14	D0	D7	D14	D0	D7	D14	D0	D7	D14
LCT-0		0.51 ± 0.04 ^a^	1.04 ± 0.06 ^b^	1.62 ± 0.14 ^c^	n.d.			n.d.			n.d.		
LPAP-1		0.89 ± 0.04 ^a^	1.63 ± 0.05 ^b^	2.11 ± 0.03 ^c^	n.d.			n.d.			n.d.		
LPAP-2		1.12 ± 0.03 ^a^	1.80 ± 0.04 ^b^	3.09 ± 0.05 ^c^	n.d.			n.d.			n.d.		
LPAP-3		1.23 ± 0.02 ^a^	2.22 ± 0.02 ^b^	4.07 ± 0.02 ^c^	n.d.			n.d.			n.d.		
LBRO-1		0.90 ± 0.02 ^a^	1.84 ± 0.04 ^b^	2.15 ± 0.04 ^c^	n.d.			n.d.			n.d.		
LBRO-2		1.22 ± 0.02 ^a^	1.99 ± 0.02 ^b^	3.24 ± 0.03 ^c^	n.d.			n.d.			n.d.		
LBRO-3		1.46 ± 0.01 ^a^	2.44 ± 0.01 ^b^	4.56 ± 0.03 ^c^	n.d.			n.d.			n.d.		
Effect significance (*p*-value)	Enzyme type	<0.0001 ***	<0.0001 ***	<0.0001 ***	-			-			-		
	Enzyme concentration	<0.0001 ***	<0.0001 ***	<0.0001 ***									

Different letters within the same row indicate significant differences among storage times for the same sample according to Tukey’s HSD test (*p* < 0.05). The *p*-values for enzyme type and enzyme concentration were calculated only for enzyme-treated samples. n.d.—not detected in any sample at D0, D7 or D14. *** *p* < 0.001.

**Table 8:** The frequency of CATA attribute selection and the *p*-values of Cochran’s Q test for pork loin samples supplemented with papain and bromelain.

Attributes	Selection Frequency of CATA Attributes for Each Sample							Cochran’s Q Test *p*-Values
	LCT-0	LPAP-1	LPAP-2	LPAP-3	LBRO-1	LBRO-2	LBRO-3	
Uniform color	15	19	22	24	23	22	24	0.103
Pinkish-red color	18	18	17	13	18	18	16	0.781
Glossy appearance in cross-section	18	19	20	22	18	19	19	0.943
Fibrous appearance	12	20	21	21	21	20	21	0.101
Dry surface	18	16	15	15	17	16	17	0.973
Presence of a white layer	3	11	14	15	14	13	13	0.009 **
Characteristic meat odor	24	23	24	23	23	24	23	0.999
Spice odor	7	11	11	13	11	11	11	0.716
Smoky odor	12	19	19	21	19	18	18	0.284
Off-odor atypical of meat	7	8	9	9	8	8	8	0.996
Characteristic pork flavor	18	20	21	21	19	19	18	0.946
Spice flavor	9	10	10	10	10	10	10	1.000
Pleasant balanced flavor	21	23	23	23	22	23	22	0.993
Salty taste	16	15	15	15	16	16	15	0.999
Sour taste	9	9	8	8	8	7	8	0.994
Bland taste	8	8	4	5	5	5	8	0.588
Elastic texture	19	17	15	13	16	16	12	0.555
Tender texture	13	14	19	19	16	17	17	0.548
Homogeneous texture	23	22	22	23	23	24	24	0.993
Crumbly texture	12	13	14	14	14	14	15	0.989
Firm consistency	19	17	14	14	15	15	12	0.448
Juiciness	14	16	20	20	17	21	21	0.204
Overall acceptability	24	22	24	23	22	24	23	0.975

Cochran’s Q test was used to evaluate differences in the selection frequency of each CATA attribute among samples. ** *p* ≤ 0.01. LCT-0—control sample; LPAP-1, LPAP-2, and LPAP-3—samples treated with papain at 0.015%, 0.030%, and 0.045%, respectively; LBRO-1, LBRO-2, and LBRO-3—samples treated with bromelain at 0.015%, 0.030%, and 0.045%, respectively.

**Table 9:** Mean scores (± SD) of sensory attributes evaluated by quantitative descriptive analysis (QDA) for pork loin samples.

Sensory Attribute	Control	Papain-Treated Samples			Bromelain-Treated Samples			*p*-Value	
	LCT-0	LPAP-1	LPAP-2	LPAP-3	LBRO-1	LBRO-2	LBRO-3	F1	F2
Uniform color	6.53 ± 0.18	7.10 ± 0.21	7.43 ± 0.16	7.70 ± 0.16	7.57 ± 0.17	7.40 ± 0.13	7.73 ± 0.17	0.262	<0.0001 ***
Pinkish-red color	6.07 ± 0.21	5.93 ± 0.22	5.87 ± 0.20	5.30 ± 0.27	6.00 ± 0.20	5.97 ± 0.19	5.73 ± 0.19	0.252	0.059
Glossy appearance in cross-section	6.53 ± 0.17	6.57 ± 0.16	6.70 ± 0.15	7.00 ± 0.14	6.43 ± 0.17	6.60 ± 0.15	6.67 ± 0.15	0.139	0.109
Fibrous appearance	5.67 ± 0.19	6.80 ± 0.25	6.87 ± 0.23	6.97 ± 0.23	6.90 ± 0.19	6.77 ± 0.23	6.97 ± 0.17	1.000	<0.0001 ***
Dry surface	5.63 ± 0.21	5.37 ± 0.19	5.30 ± 0.20	5.23 ± 0.13	5.50 ± 0.18	5.43 ± 0.20	5.60 ± 0.16	0.159	0.454
Presence of a white layer	2.53 ± 0.24	3.63 ± 0.24	4.00 ± 0.31	4.17 ± 0.28	4.03 ± 0.26	3.93 ± 0.29	3.93 ± 0.27	0.880	0.000 ***
Characteristic meat odor	8.27 ± 0.24	8.23 ± 0.20	8.33 ± 0.19	8.20 ± 0.20	8.23 ± 0.15	8.37 ± 0.19	8.20 ± 0.14	0.946	0.873
Smoky odor	5.33 ± 0.14	6.23 ± 0.16	6.27 ± 0.14	6.50 ± 0.16	6.27 ± 0.14	6.17 ± 0.17	6.20 ± 0.17	0.331	<0.0001 ***
Foreign odor, atypical of meat	2.13 ± 0.17	2.33 ± 0.20	2.43 ± 0.20	2.43 ± 0.19	2.30 ± 0.17	2.33 ± 0.18	2.30 ± 0.16	0.549	0.623
Characteristic pork flavor	6.57 ± 0.13	6.73 ± 0.24	6.90 ± 0.25	6.93 ± 0.25	6.63 ± 0.16	6.67 ± 0.15	6.57 ± 0.16	0.151	0.603
Pleasant, balanced flavor	7.60 ± 0.16	7.87 ± 0.16	7.93 ± 0.17	7.87 ± 0.16	7.80 ± 0.16	7.87 ± 0.16	7.73 ± 0.16	0.503	0.431
Salty taste	5.33 ± 0.20	5.23 ± 0.18	5.20 ± 0.19	5.20 ± 0.19	5.37 ± 0.19	5.30 ± 0.17	5.20 ± 0.18	0.613	0.897
Sour taste	2.53 ± 0.15	2.50 ± 0.16	2.30 ± 0.15	2.30 ± 0.15	2.40 ± 0.15	2.27 ± 0.14	2.33 ± 0.16	0.788	0.522
Bland taste	1.93 ± 0.21	1.90 ± 0.21	1.43 ± 0.11	1.53 ± 0.12	1.57 ± 0.16	1.50 ± 0.11	1.90 ± 0.20	0.809	0.131
Elastic texture	5.60 ± 0.14	5.30 ± 0.15	5.07 ± 0.17	4.80 ± 0.18	5.17 ± 0.17	5.17 ± 0.17	4.70 ± 0.19	0.746	0.001 ***
Tender texture	5.07 ± 0.17	5.17 ± 0.17	5.80 ± 0.17	5.80 ± 0.18	5.43 ± 0.15	5.53 ± 0.15	5.57 ± 0.14	0.556	0.002 **
Homogeneous texture	8.00 ± 0.17	7.93 ± 0.15	7.97 ± 0.19	8.00 ± 0.17	8.10 ± 0.18	8.13 ± 0.18	8.14 ± 0.11	0.242	0.987
Crumbly texture	4.37 ± 0.23	4.50 ± 0.20	4.57 ± 0.20	4.53 ± 0.20	4.63 ± 0.20	4.63 ± 0.21	4.77 ± 0.19	0.386	0.879
Firm consistency	5.53 ± 0.15	5.27 ± 0.15	4.97 ± 0.17	4.90 ± 0.16	5.07 ± 0.16	5.10 ± 0.15	4.70 ± 0.19	0.502	0.008 **
Juiciness sensation	5.77 ± 0.24	6.03 ± 0.20	6.47 ± 0.16	6.57 ± 0.20	6.17 ± 0.17	6.60 ± 0.18	6.67 ± 0.18	0.429	0.002 **
Overall acceptability	8.20 ± 0.18	8.00 ± 0.20	8.27 ± 0.14	8.10 ± 0.18	7.97 ± 0.16	8.23 ± 0.13	8.03 ± 0.19	0.747	0.429

F1—enzyme type; F2—enzyme concentration. ** *p* ≤ 0.01; *** *p* ≤ 0.001. LCT-0—control sample; LPAP-1, LPAP-2, and LPAP-3—samples treated with papain at 0.015%, 0.030%, and 0.045%, respectively; LBRO-1, LBRO-2, and LBRO-3—samples treated with bromelain at 0.015%, 0.030%, and 0.045%, respectively.

## Data Availability

The original contributions presented in this study are included in this article; further inquiries can be directed to the corresponding author.
